# Mapping uncharted territory in ice from zeolite networks to ice structures

**DOI:** 10.1038/s41467-018-04618-6

**Published:** 2018-06-05

**Authors:** Edgar A. Engel, Andrea Anelli, Michele Ceriotti, Chris J. Pickard, Richard J. Needs

**Affiliations:** 1 TCM Group, Cavendish Laboratory, J J Thomson Avenue, Cambridge, CB3 0HE UK; 2 Laboratory of Computational Science and Modeling, Institute of Materials, École Polytechnique Fédérale de Lausanne, 1015 Lausanne, Switzerland; 3 Department of Materials Science and Metallurgy, University of Cambridge, 27 Charles Babbage Road, Cambridge, CB3 0FS UK; 4 Advanced Institute for Materials Research, Tohoku University, 2-1-1 Katahira, Aoba, Sendai 980-8577 Japan

## Abstract

Ice is one of the most extensively studied condensed matter systems. Yet, both experimentally and theoretically several new phases have been discovered over the last years. Here we report a large-scale density-functional-theory study of the configuration space of water ice. We geometry optimise 74,963 ice structures, which are selected and constructed from over five million tetrahedral networks listed in the databases of Treacy, Deem, and the International Zeolite Association. All prior knowledge of ice is set aside and we introduce “generalised convex hulls” to identify configurations stabilised by appropriate thermodynamic constraints. We thereby rediscover all known phases (I–XVII, i, 0 and the quartz phase) except the metastable ice IV. Crucially, we also find promising candidates for ices XVIII through LI. Using the “sketch-map” dimensionality-reduction algorithm we construct an a priori, navigable map of configuration space, which reproduces similarity relations between structures and highlights the novel candidates. By relating the known phases to the tractably small, yet structurally diverse set of synthesisable candidate structures, we provide an excellent starting point for identifying formation pathways.

## Abstract (web summary)

Ice is one of the most well-studied condensed matter systems, yet new phases are still being discovered. Here the authors report a large-scale computational study of the configuration space of water ice, creating a navigable “sketch-map” including new predicted phases as well as relationships between different structures.

## Introduction

Ice is a complex system of interest across much of science, ranging from astrophysics to biology. On the Earth’s surface and in its atmosphere, it plays a central role in determining climate and in countless natural processes and technological applications. Ice is also a key constituent of the Earth’s crust and mantle. Its phase diagram and properties have been investigated across a wide range of temperatures and pressures by experimentalists and theoreticians alike.

A total of 18 crystalline ice phases have been formed under various conditions^1^, 7 of which are metastable^2^. In addition, a number of hypothetical ice phases^3–14^ have been predicted and characterised using computer simulations. All of these phases are molecular crystals that fulfil the “Bernal–Fowler ice rules”^15^ and form four-connected networks. In most ice phases, the distinct ways of dressing the oxygen sublattice with hydrogen atoms within the ice rules (the so-called “proton-orderings”) are quasi-energetically degenerate^16^. Theoretical studies have also suggested structures of water ice under ultrahigh pressures of up to many terapascals and its eventual decomposition^17,18^.

The phase diagram of ice has recently received renewed interest, first, because the theoretical discovery of the s-III clathrate hydrate^13^ and the experimental description of ice XVII^19^ and of two-dimensional forms of ice^14,20,21^ have demonstrated that our understanding of ice is far from complete. Second, it has become apparent that the nucleation and melting of ice are complex processes in which metastable ice phases play a role^22,23^. In classical nucleation theory, an interfacial free energy advantage of a few percent will lead to preferential nucleation of metastable phases with free energies up to around 10 meV/H_2_O above the stable phase^24^.

Despite valiant efforts using structure searching methods such as ab initio random structure searching (AIRSS)^25^, to our knowledge no comprehensive study of (meta)stable ice phases and their formation has been published to date. The problem is two-fold: first, the enormous configuration space must be explored in a reasonably comprehensive manner. Second, in order to render the structure search relevant to experiment, the large number of theoretical (meta)stable structures generated in the process must be reduced to those that can be formed experimentally. This refinement must be a priori and quantitative. Finally, different stabilising factors—such as the absorption of guest molecules^26^—can be investigated further, and methods such as forward flux sampling^27–30^ and enhanced sampling metadynamics^31–33^ may be used to identify possible synthetic pathways.

This work aims for a comprehensive study of crystalline ice phases, focussing on the exploration of configuration and the reduction of the resulting intractably large amount of structure data to a small number of structures, which are likely to be accessible experimentally. In the first section of the Results we exploit the isomorphism between ice and silica networks^12^ to explore the relevant parts of the configuration space of ice using databases of theoretically enumerated, four-connected networks. In the second section of the Results we rationalise the resultant structural data on the basis of purely energetic considerations and thereby identify structures that can be stabilised under pressure. By design, this approach cannot identify structures stabilised by thermodynamic and kinetic constraints other than pressure, such as temperature, electric fields, concentrations of guest molecules, etc. In the third section of the Results we overcome this limitation by developing a “generalised convex hull” (GCH) construction. Moreover, we use the sketch-map algorithm^34^ to construct a navigable map of the configuration space of ice. This primarily serves as an aide in developing an intuitive understanding of structural relationships. However, it also shows potential for helping to identify formation pathways for new candidate ice phases.

## Results

### Exploring configuration space

The strong isomorphism between ice and silica networks has previously been explored in ref. ^12^ and arises because both silica and water preferentially form four-connected networks composed of corner-sharing tetrahedral units. The basic building blocks of silica and water ice are so similar that it is even possible to form silica/water hetero-networks in which silicate oligomers form part of the hydrate lattice^35,36^.

**Fig. 1:**
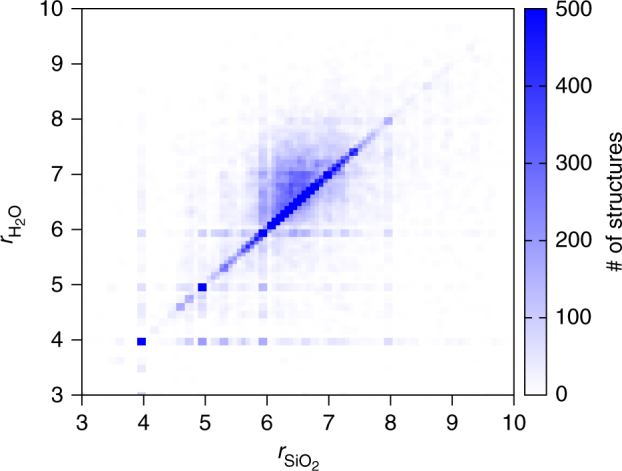
Isomorphism between SiO_2_ and H_2_O polymorphs. Correlation between the average ring sizes, *r*, of SiO_2_ and H_2_O polymorphs. More than 1/3 of the ice polymorphs retain the ring statistics of their counterpart SiO_2_ network

There is a vast literature on four-connected structures, including an atlas describing the underlying networks of porous crystalline zeolites^37^ and a number of very large databases of theoretically enumerated networks, such as the databases of Treacy^38^ and Deem^39^. Graph network enumeration has previously been applied to crystal structure prediction^40^ and, in particular, to *sp*^2^- and *sp*^3^-carbon^41,42^. The above databases have proven to be a valuable resource in searching for *sp*^3^ allotropes of carbon^43^ and constitute a comprehensive source of four-connected networks from which topologically distinct phases of ice can be constructed and geometry optimised to the respective associated local minimum energy structures using conjugate gradient methods. Recently, the search for computationally stable ultralow-density ices^44^ on the basis of the atlas of zeolites^37^ has hinted at the potential of this approach, despite its more limited scope and despite only considering stabilisation under (effective negative) pressure. The search for (meta)stable ice phases is facilitated by the strong correspondence between zeolite and ice structures. Figure 1 shows the strong correlation between the average ring sizes of SiO_2_ structures and their counterpart H_2_O polymorphs after geometry optimisation, indicating that structurally distinct SiO_2_ networks generally translate into structurally distinct H_2_O networks.

The large size of the databases of hypothetical zeolites necessitates some preselection of structures. Tribello et al.^12^ show that the energies and densities of low-density SiO_2_ networks and their counterpart H_2_O networks are correlated, but this correlation does not carry across to structures of densities comparable to and higher than that of ice Ih (see Supplementary Note 1 and Fig. 1). Consequently, neither SiO_2_ lattice energies nor densities can be used for preselection. Since all known ice phases (with the exception of ice V/XIII) have unit cells containing no more than 16 molecules, applying a cutoff to the unit cell size provides a reasonable method for preselection, which can be improved systematically by including structures with larger unit cells. In practice, we preselect only networks with unit cell volumes of no more than 800 Å^3^ and without 3-rings, which would normally induce excessive strain in an ice structure. Out of the 331,172 (Deem) and 5,389,408 (Treacy) zeolites, this leaves 74,731 structures. This selection contains duplicates since the databases are not mutually exclusive. Low-density structures with low SiO_2_ lattice energies are added back in by including the experimentally synthesised zeolite networks from the IZA database^45^ (see also Supplementary Fig. 2).

Geometry optimisation of the resulting 74,963 structures using first-principles quantum-mechanical methods is viable. However, at this stage only rough lattice energies are required to identify the low-energy sectors of configuration space. The definition of low energies is provided by the differences between the lattice energies of different proton-orderings and between the quantum vibrational corrections of different structures, which are both of the order of 10 meV/H_2_O^46^. Benchmarking against more accurate density-functional-theory results using the PBE^47^ exchange-correlation functional (PBE-DFT) (see Supplementary Note 5), which are further benchmarked against results obtained using the rPW86-vdW2 functional^48^ in Supplementary Note 6, shows that energies from ReaxFF force fields^49^ are sufficient for this purpose (see Supplementary Fig. 5). After removing high-energy configurations, the geometries of the remaining structures are refined using PBE-DFT. Removing duplicates leaves 15,869 distinct structures.

### Phase stability and characterisation of structures

The large pool of candidate structures highlights the central challenge of computational structure searches: the number of theoretical (meta)stable configurations that can be constructed increases exponentially with system size, but only those that can be observed experimentally are of interest. Their selection must take into consideration the uncertainty in the computational framework, the possibility of kinetic and/or surface effects promoting the formation of metastable phases and the (de)stabilisation of phases by different thermodynamic boundary conditions, such as pressure.

**Fig. 2:**
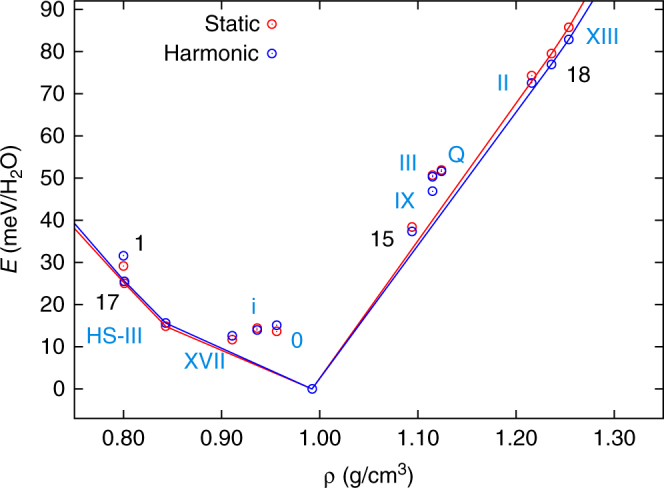
Energy-density convex hull. PBE-DFT static lattice energies (red) and free energies including harmonic vibrations (blue) relative to ice Ih for known ice phases (blue labels) and energetically competitive phases (black labels). The labels of the novel energetically competitive phases correspond to the numbering scheme in Fig. 3. The energy-density convex hulls at the static lattice and harmonic vibrational levels are indicated by red and blue solid lines, respectively

To identify the polymorphs that are most likely to form at different pressures, we first consider a well-established approach based on a convex-hull construction. The convex hull of energy (as a proxy for free energy) as a function of density, *E*_ch_(*ρ*), is formed by structures that are stable against decomposition into two or more structures with lower average energy at the same average density and the so called “tie lines” that connect them. In the absence of kinetic effects, the only phases that can be observed by manipulating the density of the system (for example through pressure) are exactly those that constitute the vertices of the energy-density convex hull (see Fig. 2). In analogy with the Bell–Evans–Polanyi principle, which states that highly exothermic chemical reactions have low activation energies, the stability of a given metastable structure can be assessed by the free energy of decomposition into stable structures. We refer to this as the “dressed energy”. Plainly put, the proximity of a metastable structure to the convex hull is a measure of its stability. The “dressed energy” is calculated by subtracting the convex-hull energy at the corresponding density *ρ* from the lattice energy *E* (as a proxy for free energy), *E*_dr_ = *E* − *E*_ch_(*ρ*). Based on *E*_dr_, we refine the selection of ice structures as specified in Computational methods. Ultimately, only structures with *E*_dr_ less than 10 meV are retained, for which kinetic, entropic and/or surface effects may plausibly lead to preferential formation during nucleation.

Setting aside all prior knowledge of ice, this procedure identifies the theoretical i, 0 and quartz phases and the known Ih/XI, II, III/IX, V/XIII, VII/VIII and X phases of ice. Moreover, it identifies the structure that has since been identified experimentally as the porous ice XVII^19,50^. This clearly demonstrates the potential of our structure searching approach. However, not all known ice phases are classified as synthesisable, which highlights the limitations of the established convex-hull approach: it fails to identify synthesisable metastable structures (such as ice IV and XII/XIV) and structures that can be stabilised and made synthesisable by thermodynamic and kinetic constraints other than pressure (such as XVI that initially forms by absorption of H_2_ guest molecules). These limitations will be addressed in the following.

In addition, the ice counterparts of the zeolites with network codes IRR, IWV, SGT and DDR and three hypothetical zeolites are identified as prime candidates for stabilisation by varying the system density. The counterparts of the IRR and IWV (not shown in Fig. 2), 207_1_4435 and DDR zeolites (labelled 1 and 17 in Fig. 2) are excellent candidates for stabilisation under negative pressure or by inclusion of guest molecules. The DDR counterpart, in particular, has previously been proposed as a possible clathrate hydrate^12^. The IRR and IWV counterparts exhibit substantially lower densities than the known CS-I, CS-II and HS-III clathrate hydrates^51–54^, suggesting that they may only become stable at large negative pressures. Conversely, the counterparts of the PCOD8172143 and 11_2_15848 zeolites (labelled 15 and 18 in Fig. 2) may be stabilised under positive pressure (also see Supplementary Fig. 4).

When comparing structures whose stabilities lie within a few meV/H_2_O of each other, anharmonic quantum nuclear effects (QNE) must be accounted for, as highlighted by the stabilisation of ice Ih with respect to Ic by anharmonic quantum nuclear vibrations^46^, as well as by effects of similar magnitude observed in other H-bonded crystals^55^. Anharmonic QNE in particular stabilise ice XVII and the HS-III clathrate by a few meV/H_2_O with respect to Ih (see Table SIII). Their resultant zero pressure free energies exceed that of Ih by only 6.8 and 7.8 meV/H_2_O at the PBE-DFT level, respectively. The relative stability of the counterparts of the PCOD8172143 and 11_2_15848 zeolites with respect to Ih, on the other hand, is affected very little (see Supplementary Note 4 for further detail).

### Using machine-learning to navigate the structural landscape

An analysis based on the energy-density convex hull as in the second section of the Results identifies candidate structures that can be stabilised by pressure. However, this does not address several crucial issues: (a) obtaining a global picture of configuration space from which one can gather an intuitive understanding of the relations between different polymorphs; (b) assessing the effectiveness of the structure search, identifying more or less obvious “gaps”; and (c) selecting candidates stabilised by thermodynamic constraints other than pressure, such as absorption of guest molecules, electric fields, etc. All of these problems can be tackled effectively within a framework that borrows ideas from the machine-learning community. Points (a) and (b) are addressed by constructing an abstract, unbiased and general two-dimensional representation of configuration space in terms of the similarity relations between structures. Point (c) is addressed by generalising the conventional convex hull construction.

The first key ingredient of an intuitive representation of configuration space is a measure of the similarity of different configurations. We use the smooth overlap of atomic positions (SOAP) kernel^56^ combined with an entropy-regularised matching (REMatch) approach^57^. This captures the fundamental symmetries of the problem, such as invariance to alternative representations of the same periodic structure, particle labelling and rigid rotations and translations of the atomic coordinates. Based on the kernel-induced distance, we apply the sketch-map algorithm^34^ to obtain a two-dimensional representation that reproduces as accurately as possible the (non-linearly transformed) distance between each pair of structures. The construction and its parameters were designed to assess the oxygen lattice while being insensitive to proton-disorder and hydrogen-bonding defects.

**Fig. 3:**
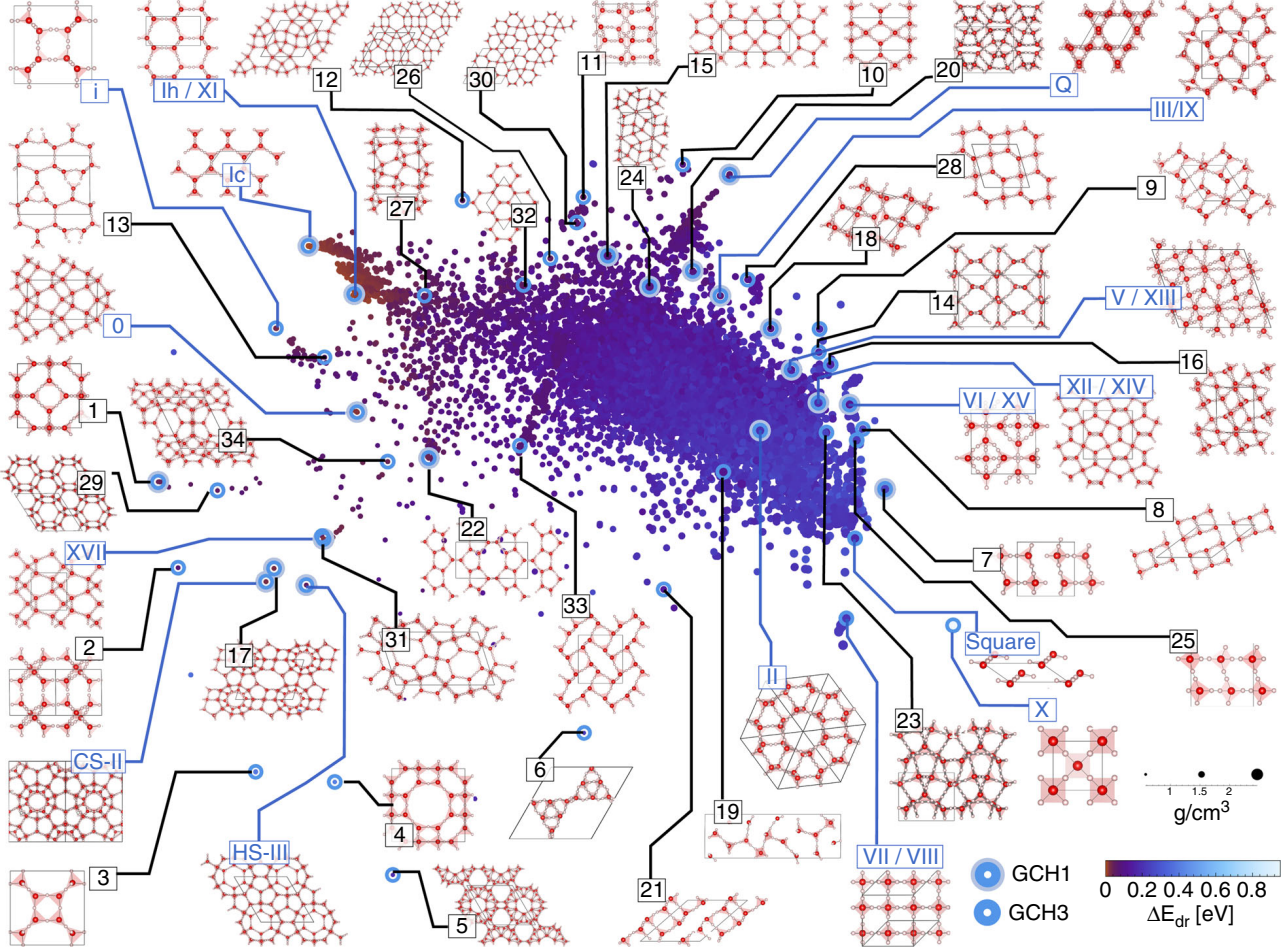
Sketch map of the structural similarity of 15,869 distinct PBE-DFT geometry-optimised ice structures. The sketch-map coordinates correlate strongly with density and configurational energy but ultimately measure abstract structural features, which leaves their numerical value without intuitive meaning. They are therefore not shown. Instead the density and static lattice energy of each structure is encoded by the size and colour of the respective point on the map. Known ice phases are labelled in blue. The 34 new candidates are labelled in black and numbered in order of increasing dressed energy relative to the GCH3. Their atomic structures are shown to highlight their structural diversity

The resulting map is shown in Fig. 3 and provides a much-needed global picture of the lie of the land. Notably, it is spanned by collective coordinates measuring abstract structural features, which in general cannot be related to single conventional observables such as density in a meaningful way. Consequently, their numerical values are not shown in Fig. 3.

Several observations highlight the heuristic value of such a representation: (1) The positions on the map correlate well with both density and lattice energy (see Supplementary Note 2 and Supplementary Fig. 5); (2) Structures related by proton-disorder, such as Ih/XI, III/IX and VII/VIII, are clustered together; (3) Structures related by stacking disorder, such as Ih, Ic and Isd, are clustered together; (4) The spread in energy at a given point on the map is comparable to the energy scale of stacking defects and H-bonding defects. H-bonding defects and different proton-orderings develop during the geometry optimisation of the ice structures, which (in analogy with their SiO_2_ parent structures) are initialised with bond-centred protons.

Furthermore, the general structure of the map is consistent with the strategy we followed to construct our set of structures. The upper portion of the map, corresponding to tetrahedral ices and silica-like networks is densely sampled, with structures clustered in partially overlapping regions. The lower part of the map corresponding to very dense (e.g., ice X) and very open structures (e.g., those originating from the IZA zeolite data set) is sparse. At high density, this sparsity results from the increasing importance of geometric constraints, which limit structural diversity and prohibit the formation of energetically feasible “mixed phases” containing structural patterns from two or more low-energy configurations. At low-density, our preselection strategy leads to sparse sampling. Sketch map therefore provides indications of the quality of configuration-space sampling, which can be used to focus the structure search on the regions that need it most.

Finally, structures with low *E*_dr_ are projected onto the periphery of the map (see Supplementary Fig. 4c), whereas the central region is largely populated by defective, “mixed phase” structures that lie far from the energy-density convex hull. This suggests that a machine-learning-inspired analysis of structures may be used to establish a GCH, which identifies configurations that can be stabilised (and made “synthesisable”^58^) by the application of appropriate thermodynamic constraints.

We define this GCH construction in analogy with the conventional energy-density convex hull in the second section of the Results, but instead of considering *E* as a function of *ρ*, we consider *E* as a function of *n* variables measuring abstract structural features, *E*(*ϕ*_1_, …, *ϕ*_*n*_). The simplices of the GCH thus correspond to structures that are stable with respect to decomposition at a given set of these abstract structural features, rather than at a particular density. The conventional energy-density convex hull allowed the identification of structures, which can be stabilised by pressure, because pressure allows the manipulation of the density of an ice sample. Conversely, the GCH allows us to identify structures, which can be stabilised by imposing thermodynamic and/or kinetic constraints that couple to the abstract structural features. In analogy with *E*_dr_, one can then define a generalised dressed energy $E_{{\mathrm{dr}}}^{(n)}$ that quantifies the stability of a given configuration subject to constraints that couple to the *n* structural features *ϕ*_1_ … *ϕ*_*n*_.

In practice, a kernel principal-component analysis (KPCA)^59^ is performed to extract the KPCA descriptors *ϕ*_*i*_, which encode the key structural features and can be sorted in order of decreasing importance. Crucially, the KPCA components (unlike the collective variables defining the highly non-linear sketch-map projection) form a vector space in which the notion of convexity is well defined.

The GCH construction provides a powerful tool for discovery, since one can select configurations that are both low in energy and “extremal” in the sense of structural features described by one or more KPCA descriptors. By increasing the number *n* of features considered, the screening becomes progressively more inclusive, since multiple axes of structural diversity are considered simultaneously. In practice, the selection was further refined by (automatically) eliminating structurally related configurations, as discussed in Supplementary Note 2.

Including three KPCA descriptors in the GCH construction, we identify 50 structures within 20 meV of the GCH (see Fig. 3), which include all of the known ice phases except ice IV. Ice IV is not classified as synthesisable due to its particularly high lattice energy, which is consistent with the experimental observation that ice IV is metastable and only forms occasionally upon slow heating of high-density amorphous ice before annealing to ice III, V or VI.

The 50 structures also include the theoretical i, 0, quartz and square phases, the CS-II clathrate hydrate (which is identical in structure to ice XVI) and the HS-III clathrate hydrate. Furthermore, we identify 34 new configurations that are excellent candidates for experimental formation and that we propose as candidates for ices XVIII through LI. Among them are, in particular, the ice counterparts of the DDR, SGT and NON zeolites, which were previously suggested as promising candidates for clathrate hydrates by Tribello et al.^12^, and two structures reminiscent of a high pressure structure with *Pbcm* symmetry proposed by Hermann et al.^60^. Notably, while the most promising candidates for experimental formation (as indicated by their ordering in Fig. 3) are low-density ice counterparts of different zeolite networks, the counterpart of the ITT network, which was suggested as the most stable “aeroice” structure below around −0.4 GPa in ref. ^44^, is dynamically unstable at the employed level of theory. For reference, using the rPW86-vdW2 exchange-correlation functional ITT ice is still much less stable than IRR ice proposed as stabilisable in this work (see Supplementary Fig. 1b). It is worth noting that the counterpart of the LTA zeolite (structure 4 in Fig. 3) has also most recently received attention as an “ultralow” density clathrate ice in ref. ^61^. While the GCH generally depends on the kernel, our choice of kernel representation is very general and rather unbiased, which is reflected by the weak dependence of this selection of structures on the choice of hyperparameters for the SOAP-REMatch kernel. Notably, the GCH is also remarkably insensitive to the details of the underlying (free) energy calculations. As shown in Supplementary Note 6, 37 out of the 38 structures are still identified as GCH vertices when the lattice energies of the structures highlighted in Fig. 3 are computed using the dispersion-corrected rPW86-vdW2 exchange-correlation functional^48^ instead of the PBE functional, despite significant differences with respect to the PBE lattice energies. The rPW86-vdW2 functional has been shown to be particularly accurate for the known phases of ice^62^. In contrast, the energy-density CH depends more sensitively on the choice of exchange-correlation functional.

## Discussion

The success of the GCH construction in discovering the known ice phases and clathrate hydrates entirely a priori highlights that, although kinetic factors play an important role in determining which ice phases are formed in practice, structural and simple energetic considerations can provide a great deal of physical insight. More importantly, it demonstrates that the GCH approach does not simply discern structurally diverse configurations, but very effectively selects configurations that can be formed in experiment. It thereby provides strong support for the 34 proposed, new, structurally diverse candidates for ices XVIII–LI. This should spur experimental efforts to ratify our predictions.

At this stage, the candidates are embedded in a human-readable sketch map of configuration space mainly as an aide in developing an intuitive understanding of the relation of the proposed candidates to the known ice phases. However, highlighting the phase transitions between the known ice phases suggests that proximity on the sketch map is a good indicator for the existence of a viable transition pathway. In conjunction with the GCH construction, the sketch map therefore provides a tractably small and yet structurally diverse set of synthesisable candidate structures and a means of identifying end points and suitable reaction coordinates for further investigation of formation pathways, for example, using transition state sampling^63^, umbrella sampling^64^, forward flux sampling^27^ or enhanced sampling metadynamics approaches^65^, which have already proven successful in simulating the nucleation of ice^28–32^. The relation of the KPCA descriptors in the GCH construction to conventional quantities, such as density, vibrational spectra and concentrations of different types of guest molecules, promises to provide more direct guidance in identifying experimental formation pathways. However, this goes beyond the scope of this study.

In addition, the approach demonstrated in this work sheds light on the energetics of proton-order/disorder and stacking-disorder, as well as H-bonding and planar defects, and also provides a glimpse of the preferred (quasi-) two-dimensional forms of ice.

In its current state, the biggest limitation of our structure search is the preselection cutoff on system size. Relaxing this cutoff will drive the structure search towards completeness, which is the obvious next step. More generally, the connection between structural patterns and configurational energy exposed by the sketch-map dimensionality reduction suggests an expedient recipe for even more extensive database-driven searches. These need not be limited to crystalline water ice but could range from other tetrahedrally coordinated systems, such as silica or the carbon allotropes, to liquid, disordered and glassy systems.

## Methods

### Geometry optimisations

Ice structures were initially geometry optimised using ReaxFF force fields^49^ as parametrised by Raymand et al.^66^ and implemented in the Gulp package^67^ until the energies and forces were converged to within 10^−4^ eV and 10^−3^ eV/Å, respectively. This choice is motivated and justified in Supplementary Note 5.

First-principles quantum-mechanical geometry optimisations were performed using semi-local PBE-DFT^47^ as implemented in the Castep package^68^. The choice of density functional is discussed in detail in Supplementary Note 6. The initial PBE-DFT geometry optimisations were performed with an plane-wave energy cutoff of 490 eV, Monkhorst-Pack **k**-point grids of maximum spacing of 2*π* × 0.07 Å^−1^, and on-the-fly generated ultrasoft pseudopotentials. The structures in the second section of the Results were further refined using PBE-DFT calculations with norm-conserving pseudo-potentials and, first, a plane-wave energy cutoff of 490 eV and Monkhorst-Pack **k**-point grids of maximum spacing of 2*π* × 0.07 Å^−1^, then a cutoff of 800 eV and Monkhorst-Pack **k**-point grids of maximum spacing of 2*π* × 0.04 Å^−1^, and finally a cutoff of 1200 eV and Monkhorst-Pack **k**-point grids of maximum spacing of 2*π* × 0.04 Å^−1^. The resulting energy differences between frozen-phonon configurations, atomic positions and residual forces were converged to within 10^−4^ eV/H_2_O, 10^−5^ Å and 10^−4^ eV/Å, respectively (also see Supplementary Note 3).

Harmonic vibrational modes and frequencies were calculated using a finite displacement method. Anharmonic vibrations are calculated using the vibrational self-consistent field approach described in ref. ^69^. The 3*N*-dimensional BO energy surface (where *N* is the number of atoms in the simulation cell) was described by mapping one-dimensional (1D) subspaces along the harmonic normal mode axes up to large amplitudes of four times the harmonic root-mean-square displacements, where anharmonicity is important. The 3*N*-dimensional BO surface was then reconstructed from the 1D subspaces. The 1D energy surfaces were fitted using cubic splines The anharmonic vibrational Schrödinger equation was solved expanding the vibrational wave function in terms of simple harmonic oscillator eigenstates. The inclusion of 25 states for each vibrational degree of freedom was found sufficient to obtain converged results.

Duplicates were identified by applying the “crysim” tool from the AIRSS method^25^ to the oxygen sublattices.

### Machine-learning analysis of structural relations

To assess the structural similarity between the configurations in the database under study, we used a REMatch-SOAP kernel, as implemented in the glosim.py package (http://cosmo-epfl.github.io), with the following choice of hyperparameters controlling the description of atomic environments:

/src/glosim/glosim.py -n 9 -l 6 -c 5 -g 0.5 –periodic –nocenter 1 –kernel rematch –gamma 0.01 –nonorm

Hydrogen atoms were included in the definition of the atom-density overlap but were not considered as environment centres, so as to de-emphasise proton-(dis)order in the definition of structural similarity. The choice of cutoff radius was tuned to achieve a clear separation between the known phases of ice in the database.

The non-linear sketch-map dimensionality reduction scheme (http://sketchmap.org) was then applied to the SOAP kernel measure of similarity for 400 farthest-point-sampled landmark structures following the procedure described in ref. ^70^ and using the following parameters: *σ* = 0.12, *A* = 2, *B* = 4, *a* = 2, and *b* = 2.

### Data availability

The data that support the findings of this study are available at the following 10.24435/materialscloud:2018.0010/v1.

## Electronic supplementary material

Supplementary Information

Supplementary Data 1

Description of Additional Supplementary Files
