# Supplementary material for: Mapping uncharted territory in ice from zeolite networks to ice structures
Source: Nat Commun. 2018 Jun 5;9:2173. doi: 10.1038/s41467-018-04618-6 (PMC5988809; doi:10.1038/s41467-018-04618-6)
Supplement: Supplementary file 1 — Supplementary Information [file 41467_2018_4618_MOESM1_ESM.pdf]

Supplementary Material: Mapping uncharted territory in ice

Edgar A. Engel,[1] Andrea Anelli,[2] Michele Ceriotti,[2] Chris J. Pickard,[3,4] and Richard J. Needs[1]

[1] *TCM Group, Cavendish Laboratory, J J Thomson Avenue, Cambridge, CB3 0HE, UK*
[2] *Laboratory of Computational Science and Modeling, Institute of Materials, École Polytechnique Fédérale de Lausanne, 1015 Lausanne, Switzerland*
[3] *Department of Materials Science and Metallurgy, 27 Charles Babbage Road, Cambridge CB3 0FS, UK*
[4] *Advanced Institute for Materials Research, Tohoku University, 2-1-1 Katahira, Aoba, Sendai 980-8577, Japan*

(Dated: April 20, 2018)

(a) ReaxFF (left) and PBE-DFT (right) H_2O static lattice energies versus SiO_2 static lattice energies.

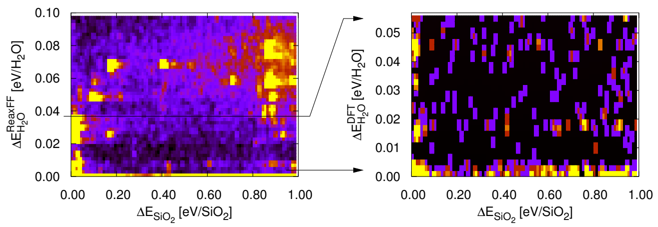

(b) ReaxFF (left) and PBE-DFT (right) H_2O static lattice energies versus SiO_2 densities.

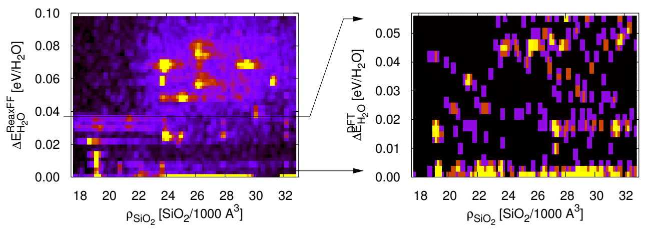

Supplementary Figure 1. Weak correlation between the static lattice energies and densities of SiO_2 networks and the stabilities of the corresponding water ice structures. All energies are measured with respect to the lowest energy configuration.

Supplementary Note 1. SELECTION OF LOW ENERGY STRUCTURES

Supplementary Note 1.1. Preselection

The sizes of the databases of hypothetical zeolites and the correlation between the energies of low-density SiO_2 networks and their counterpart H_2O structures observed by Tribello *et al.* [1] poses the question of whether the energies and densities of the zeolite networks can be used to preselect ice structures belonging to the low energy sector of the configuration space. Supplementary Fig. 1 (a) shows that this correlation does not carry across to structures of densities comparable to and higher than Ih. There is also no significant correlation between the densities of the SiO_2 networks and the energies of their counterpart H_2O structures (Supplementary Fig. 1 (b)). Hence we preselect solely on the basis of unit cell size, which, in particular, excludes structures with very open networks, such as clathrate hydrates. Configurations with open networks and low SiO_2 energies are added back in by including the experimentally known zeolites of the IZA database. These exhibit low energies compared with the bulk of the hypothetical zeolites as shown in Supplementary Fig. 2. After transformation of the preselected structures into ice structures and subsequent optimisation using ReaxFF force fields, all ice structures within

$$35 \times (1 + 5 \times |\rho/\rho_{\mathrm{Ih}} - 1|)\,\mathrm{meV/H_2O} \qquad (1)$$

of the $\rho - E_{\mathrm{st}}$ convex hull (CH), i.e., structures below the selection cut indicated by a solid black line in Supplementary Fig. 3, were retained. Duplicates – including different proton-orderings of the same phase – were removed. The selection cut is increasingly loose for structures with densities that differ from Ih, for which ReaxFF energies become increasingly inaccurate.

The selection cut is justified more generally as follows. In SiO_2 the oxygen ions are equidistant to their nearest-neighbour silicon ions, so that the protons in the counterpart ice structures are initialised at bond-centred positions. Symmetry is then broken naturally during geometry optimisation, thereby selecting an (arbitrary) particular proton-ordering. Studies of proton-ordering energetics [2, 3] and proton-ordering transition temperatures of no more than 126 K suggest that different proton-orderings of the same phase generally do not differ by more than about 10 meV/H_2O. At the same time, the vibrational free energies of (dissimilar) ice structures only differ by a few meV/H_2O (see Supplementary Table II) due to the strong molecular crystalline nature of the ice phases. This motivates neglecting proton-ordering and quantum nuclear effects (QNE) in the search for (meta-)stable phases, and simply interpreting them as a source of uncertainty in the configurational energies of the geometry optimised structures. Selection cut-offs for static lattice energies therefore need to be substantially larger than 10 meV/H_2O in order not to bias the structure search.

The selection is further justified by the observation that, out of 2599 structures with unit cell volumes up to 300Å^3, only six with PBE-DFT energies within 75 meV/H_2O of the energy-density CH are incorrectly eliminated, while 1008 are correctly retained.

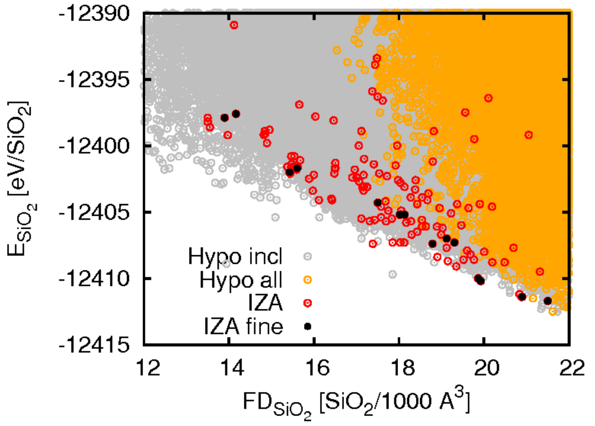

Supplementary Figure 2. Energy versus density of SiO_2 structures. The eliminated and preselected hypothetical structures from the Deem database are shown in grey and orange, respectively. The known structures from the IZA database and the subset that translates into low-energy ice structures are shown in red and black, respectively. The IZA structures exhibit below average energies. Structures forming low-energy ice structures exhibit particularly low energies, which demonstrates the correlation between the SiO_2 and H_2O energies for low-density networks observed in Ref. [1].

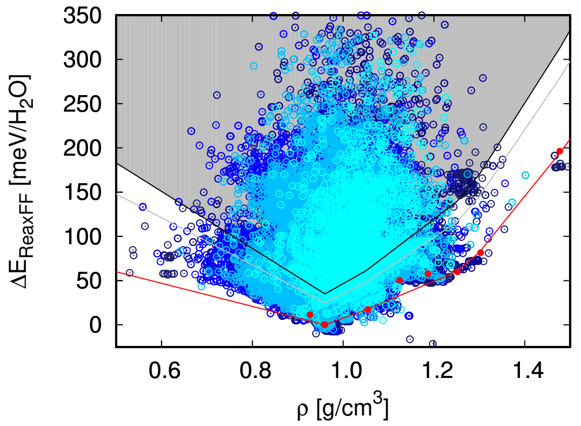

Supplementary Figure 3. Energy versus density of the ReaxFF geometry optimised ice structures. The data are coloured according to unit cell size, with lighter colours corresponding to smaller unit cells. Solid lines indicate the CH formed by the known phases of ice (red), the applied selection cut (black), and a tighter trial selection cut (grey).

Furthermore, tightening the selection cut demonstrates *a posteriori* the leniency of the applied selection cut. All unique configurations within 20 meV of the CH at the highest level of theory survive the tighter selection cut (shown as solid grey line in Supplementary Fig. 3).

Supplementary Note 2. MACHINE-LEARNING ANALYSIS

Supplementary Fig. 5 shows the strong correlation of the sketch-map collective variables with the densities and energies of the ice structures, respectively.

Supplementary Table 1 summarises the structural and energetic details of the structures highlighted on the sketch-map in Fig. 3 of the main text.

Supplementary Note 2.1. Generalised Convex Hull and Clustering Scheme

Following the generalised convex hull (GCH) procedure detailed in the main text, increasing the number of kPCA components produces an increasingly inclusive selection. Supplementary Fig. 6 shows GCH selections with one and three components, setting a cutoff threshold on the dressed energies at $20\,meV$. Points below this threshold are coloured according to their GCH dressed energies.

In both cases the number of structures with low dressed energies is large, since the database includes configurations that are only distinguished by the presence of proton disorder or stacking faults, which are expected to change the binding energy by just a few meV/H_2O. While it would be possible to inspect structures manually to identify those that are genuinely structurally distinct, it is easier to further reduce the number of proposed phases by an additional clustering step, based on the high dimensional similarity matrix. The DBSCAN algorithm implemented in the `scikit-learn` package is used to cluster the phases in classes that show a strong structural similarity. The reason for choosing this algorithm is twofold: firstly, the points are distributed in dense islands and, secondly, DBSCAN automatically detects outliers as noise, which in this context identifies clearly-distinct CH vertices. The parameters chosen for the DBSCAN step are selected based on the histogram of the distances induced by the SOAP kernel, and are chosen as follows. We used the kernel distance as a metric, set $\epsilon = 0.059$ and the minimum number of points to three.

The DBSCAN procedure is based solely on structural similarity. However, we still account for energetics by selecting one (or more) representative configurations from each cluster, running a single kPCA component GCH on each of them, and selecting only the elements falling 0.001 meV from the cluster's hull. The representative structures are then proposed by joining the list of the points detected as noise with the centers found in the

Index	Original label	H_2O/unit cell	Space group	ρ [g/cm^3]	ρ_{ref} [g/cm^3]	E_{dr}^{ρ}	E_{dr}^{GCH-1}	E_{dr}^{GCH-3}
1	207_1_4435	12	$Im\bar{3}m$	0.965	(0.772)	0.00	19.64	0.00
2	12_2_29187	6	$I4/m$	0.998	0.797	38.13	44.20	0.00
3	ACO	16	$Im\bar{3}m$	1.109	(0.887)	364.22	338.53	0.00
4	LTA	24	$Pm\bar{3}m$	0.933	(0.764)	349.61	341.04	0.00
5	BSV	48	$Ia\bar{3}d$	0.879	(0.703)	127.77	131.70	0.00
6	169_2_7915	12	$P6_122$	0.848	(0.678)	155.48	141.81	0.00
7	53_3_726600	16	$Cmcm$	1.682	(1.346)	141.80	0.00	0.00
8	20_2_26425	6	$P2_1$	1.592	(1.274)	165.75	58.47	0.00
9	12_2_32449	6	$C2/c$	1.577	(1.262)	100.96	65.68	0.00
10	84_2_1419	6	$P4_2/m$	1.349	1.089	57.14	37.74	0.00
11	61_2_8842	16	$R3$	1.339	1.085	26.36	20.96	0.00
12	169_2_10608	12	$C222_1$	1.177	0.946	50.76	38.10	0.00
13	PCOD8047078	12	$P2_1$	1.131	0.920	53.84	60.63	0.00
14	67_2_1563	16	$Pbma$	1.570	1.171	88.64	51.37	0.00
15	PCOD8172143	10	$Pnn2$	1.344	1.092	19.50	9.22	1.52
16	152_2_118474	9	$P3_121$	1.599	1.270	89.78	61.98	1.79
17	DDR	40	$C2/m$	0.996	0.801	19.02	19.83	2.40
18	11_2_15848	8	$C2/c$	1.535	1.236	32.31	7.85	4.36
19	91_2_8335121	16	$P1$	1.688	(1.350)	17.15	29.05	5.44
20	PCOD8301974	16	$I4_1/a$	1.443	1.110	31.52	14.31	8.99
21	PCOD8045578	8	$R\bar{3}m$	1.422	(1.138)	83.74	69.60	10.48
22	58_2_511	12	$Cmcm$	1.112	0.908	23.09	17.57	13.41
23	151_2_4949650	9	$P3_112$	1.638	1.313	64.30	24.97	13.54
24	PCOD8007225	16	$P\bar{1}$	1.438	(1.150)	30.20	19.83	15.54
25	2_2_342692	4	$Pbnm$	1.681	(1.345)	128.00	37.64	15.76
26	PCOD8321499	18	$C222_1$	1.260	1.024	35.38	21.64	15.97
27	PCOD8047931	16	$P2_1$	1.219	0.984	40.78	36.67	17.39
28	15_2_201714	6	$Ibam$	1.472	1.068	49.18	21.46	17.76
29	MAR	72	$P2_1/m$	0.971	0.798	16.10	34.48	17.93
30	PCOD8324623	18	$P2_1$	1.323	1.081	38.29	28.75	18.20
31	SGT	32	$I4_1/amd$	0.972	0.794	9.97	23.46	19.02
32	20_2_28176	6	$C2$	1.262	(1.010)	38.23	27.73	19.19
33	14_2_48453	8	$Pmnn$	1.355	(1.084)	36.73	33.36	19.64
34	NON	22	$Fmmm$	1.050	0.860	16.09	21.19	19.64

Supplementary Table 1. Structure data for novel candidate ice phases. Columns one and two provide the mapping between the structure indices in the main text and the original labels of the corresponding four-connected networks in the databases of Treacy *et al.* [4–6] and Deam *et al.* [7] and the IZA atlas of zeolites [8]. The next columns provide the number of molecules per unit cell, the space group, and the initial and refined PBE-DFT density, ρ and ρ_{ref}. Brackets indicate values that have been estimated noting that the refined PBE-DFT densities are consistently around 20 % smaller than those from the initial PBE-DFT calculations. The last three columns contain the dressed energies relative to the CH built on the density (E_{dr}^{ρ}), a generalised convex hull (GCH) with one principal component (E_{dr}^{GCH-1}), and three components (E_{dr}^{GCH-1}). All energies are expressed in meV/H_2O.

step before. The results of the DBSCAN clustering is presented in Supplementary Fig. 7.

Supplementary Note 3. COMPUTATIONAL METHODS

Notably, the initial PBE-DFT geometry optimisations overestimate structural densities by about 20 % compared to experiment [9], TIP4P/2005 values [10], and refined PBE-DFT data (see Supplementary Table 2). Crucially, this overestimation of densities is very similar for all structures, leaving the energy-density CH and all other CH constructions unaffected. This is further supported by comparing PBE-DFT energies with their refined counterparts for the 50 structures highlighted in Fig. 3 of the main text (see Supplementary Fig. 8).

Supplementary Note 4. QUANTUM NUCLEAR MOTION

Supplementary Table 3 shows that the differences in vibrational energies between any two phases are generally no larger than a few meV/H_2O. This largely arises from the molecular crystal nature of the ice phases, which is reflected in the high frequency parts of their vibrational spectra, which in turn dominate the vibrational energies.

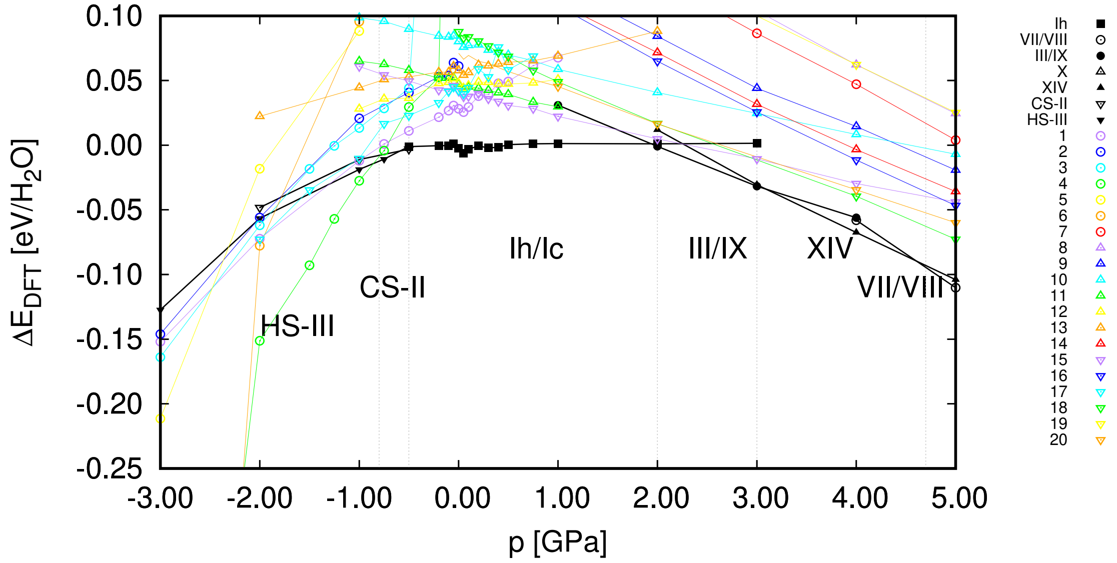

Supplementary Figure 4. Pressure dependence of the stability $\Delta E(p) \equiv E(p) - E^{\mathbf{Ih}}(p)$ of the proposed candidates. The stability regimes of the known phases of ice at the employed level of theory is shown in black for reference.

	Exp	TIP4P/2005	PBE-DFT	refined PBE-DFT	rPW86-vdW2
LTA	NA	0.61	0.93	NA	NA
SGT	NA	0.76	0.97	0.79	0.74
Ih	0.92	0.95	1.20	0.99	0.93
II	1.17	NA	1.49	1.22	1.18
III	1.14	NA	1.48	1.12	1.17
V	1.23	NA	1.57	1.26	1.24
VI	1.31	NA	1.65	1.33	1.29
XII	1.29	NA	1.61	1.30	1.27
VII	1.50	NA	1.85	1.47	1.45
VIII	1.46	NA	1.80	1.47	1.45

Supplementary Table 2. Comparison of densities of different known ice phases and proposed clathrates in g/cm^3 obtained from the initial PBE-DFT geometry optimisations with experimental [9] and TIP4P/2005 [10] data. The initial PBE-DFT geometry optimisations consistently overestimate structural densities by about 20 % compared to experimental and TIP4P/2005 data.

Nonetheless, it is vital to include accurate vibrational energies when comparing stabilities of phases which differ only by a few meV/H_2O.

Supplementary Fig. 9 shows that the harmonic and anharmonic vibrational energies of ice Ih, 0, and XVII and structure [10] converge slowly with sampling of the vibrational Brillouin Zone and thus with simulation cell size in finite displacements methods. However, the differences in vibrational energies between these ice phases converge quickly compared to the absolute vibrational energies and values, which are converged to within 0.5 meV/H_2O, can be obtained using simulation cells containing 64 H_2O molecules. Supplementary Table 4 summarises the results for ice 0, XVII and structure [10].

Supplementary Note 5. CHOICE OF FORCE FIELD

The ReaxFF [11] force field is used to preselect viable ice structures for operational and conceptual reasons. Firstly, the ReaxFF force field is a bond-ordering potential in which bonds are identified *in situ* based on the instantaneous configuration. This allows the hydrogen atoms (which are initialised in O–H–O bond-centered positions in analogy to the silicon atoms in the SiO_2 parent structures) to form a molecular crystal satisfying the Bernal-Fowler ice rules [12] during structure optimisation. In contrast, most conventional and well-proven potentials such as TIP4P/2005 [13] require molecules to be defined in advance. The use of the ReaxFF force field thus streamlines the generation of viable ice structures by shortcutting the association of hydrogen and oxygen atoms in molecules.
Secondly, the parametrisation of the ReaxFF force field used in this work [14] is not specifically optimised to reproduce the tetrahedral bonding configurations exhibited by the known phases of ice – in contrast to, for example, the TIP4P/2005 potential – arguably giving it the advantage in the context of a general exploration of the configuration space of ice (including all possible four-coordinated bonding configurations).

Supplementary Fig. 10 shows that the ReaxFF energies correlate strongly with benchmark PBE-DFT ener-

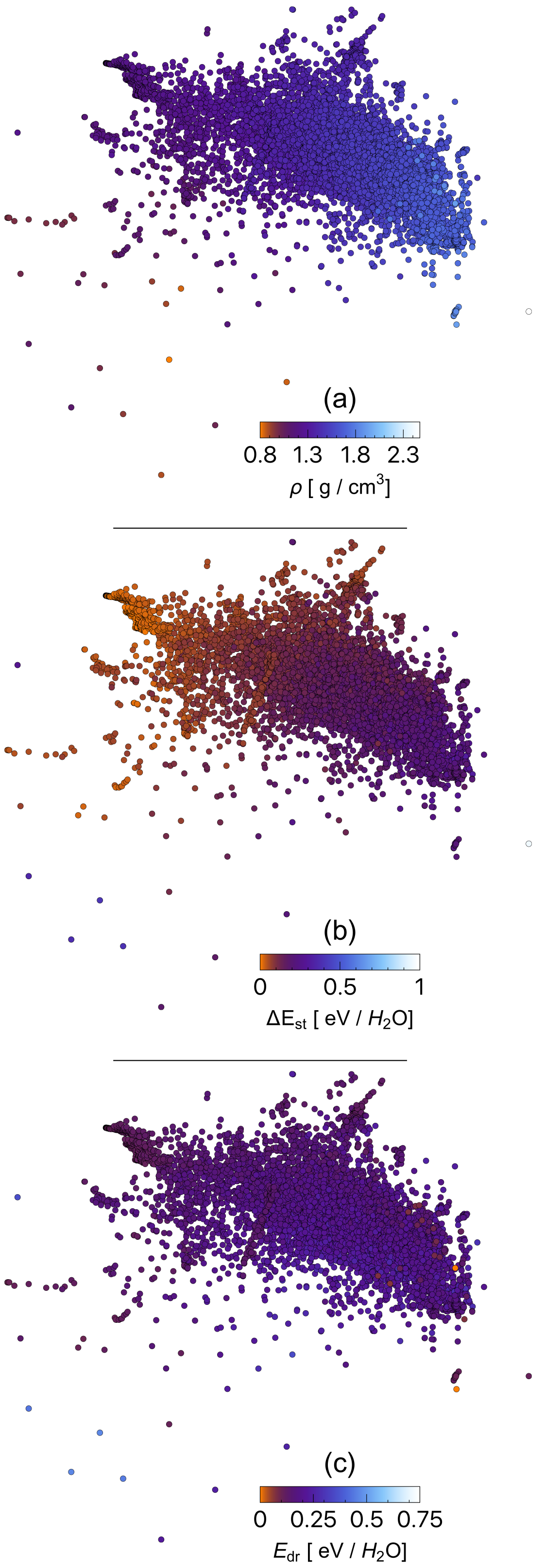

Supplementary Figure 5. Correlation of the sketch-map collective variables with energies and densities. Correlation with (a) the densities and (b) the bare and (c) dressed configurational energies of the ice structures, respectively. Here the dressed energy denotes the energy measured with respect to the energy-density CH.

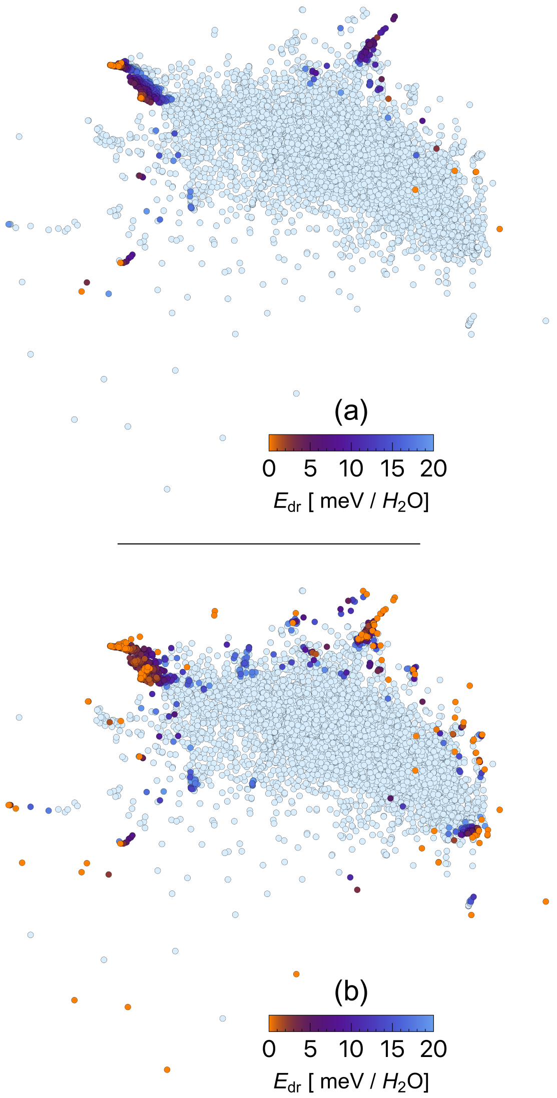

Supplementary Figure 6. Sketch-map coloured according to distance from the GCH as a measure of stabilisability. Structures are coloured according to their dressed energies with respect to the GCH constructions using (a) one and (b) three kPCA components, respectively. Structures shown in bright blue are more than 20 meV/H_2O above the hulls, while the rest is coloured according to the legends.

gies, so that an initial survey of the configuration space of ice and elimination of high energy configurations can be performed on the basis of ReaxFF data. Further to Supplementary Fig. 10, the ReaxFF force field can be shown to be a suitable choice for the preselection of viable ice structures by showing that it reproduces the relative stabilities and densities of the known phases of ice adequately well. The comparison of ReaxFF data with PBE-DFT data, as well as the calorimetry data of Whalley [9] and TIP4P/2005 force field data of Aragones *et*

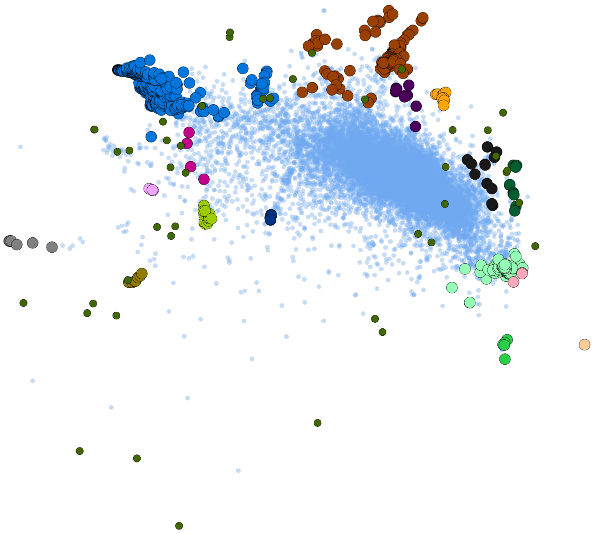

Supplementary Figure 7. Sketchmap of classes of structures identified using DBSCAN. The DBSCAN routine identifies 16 different classes which are indicated by their colouring. The smaller disks indicate the points classified as noise by the algorithm, that we consider instead to be distinct stable structures.

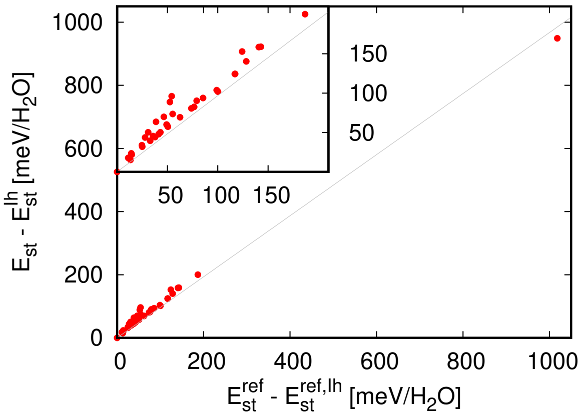

Supplementary Figure 8. Correlation between the initial ($\mathbf{E_{st}}$) and the refined ($\mathbf{E_{st}^{ref}}$) lattice energies of the known phases of ice and various other structures highlighted in Fig. 3 of the main text.

al. [15] is shown in Supplementary Tables 5 and 6. Neither the absolute energies nor the densities obtained using the ReaxFF force field are in perfect agreement with experimental and PBE-DFT values. Yet, the agreement with experimental data of Whalley [9] is comparable to that of TIP4P/2005 data [15]. More importantly, the *relative* energies and densities are sufficiently accurate for the ReaxFF energy-density CH (as constructed to preselect ice structures) to effectively reproduce the CH obtained using experimental or PBE-DFT data.

Phase	H_2O/unit cell	ρ [g cm^{-3}]	E_{ha} [meV/H_2O]	
i	8	0.94	682.9	(-0.4)
0	12	0.96	685.5	(2.1)
XI	4	0.99	683.3	(0.0)
Ih	8	0.99	683.3	(0.0)
Ic	8	0.99	683.3	(-0.1)
III	12	1.12	683.7	(0.4)
IX	12	1.12	684.0	(0.6)
Q	3	1.12	683.6	(0.3)
IV	16	1.15	—	(—)
II	12	1.22	682.3	(-1.0)
XIII	28	1.26	682.6	(-0.7)
XIV	12	1.30	681.2	(-2.1)
XV	10	1.33	678.8	(-4.5)
VI	10	1.33	678.8	(-4.5)
VII	2	1.47	684.0	(0.7)
VIII	4	1.47	(639.5)	(-43.9)
X	2	2.39	(538.0)	(-145.3)

Supplementary Table 3. Unit cell size, PBE-DFT densities, ρ, and zero temperature and pressure harmonic vibrational energies E_{ha} of the known ice phases (for simulations cells containing between 8 and 16 molecules). The E_{ha} relative to ice XI are shown in brackets. The E_{ha} have been rescaled to a simulation cell size of 12 molecules assuming that the simulation cell size dependence shown in Supplementary Fig. 9 is universal. Note that ice VIII and X are dynamically unstable at zero pressure.

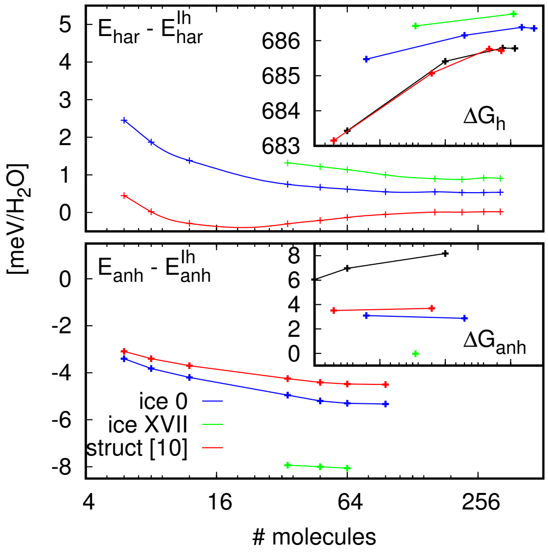

Supplementary Figure 9. Convergence of vibrational energies with simulation cell size. The harmonic (top panel) and anharmonic (bottom panel) vibrational energies, E_{har} and E_{anh}, are measured with respect to those of Ih.

Phase	ρ [g cm^{-3}]	$\Delta E_{\rm st}$	$\Delta E^{\rm TS}_{\rm st}$	$\Delta E_{\rm har}$	$\Delta E_{\rm anh}$	ΔG
XVII	0.843	14.5	23.7	+1.3	-8.0	7.8
[10]	0.911	11.4	16.8	-0.1	-4.5	6.8
0	0.956	13.3	15.8	+0.5	-5.3	8.5

Supplementary Table 4. Relative free energies including quantum nuclear motion for select structures. PBE densities, ρ, and static lattice, harmonic and anharmonic vibrational energies, $\Delta E_{\rm st}$, $\Delta E_{\rm har}$ and $\Delta E_{\rm anh}$, and free energies including anharmonic vibrations, ΔG, with respect to XI. All energies are measured in meV/H_2O and at zero temperature. PBE-TS static lattice energies, $\Delta E^{\rm TS}_{\rm st}$, are given for comparison.

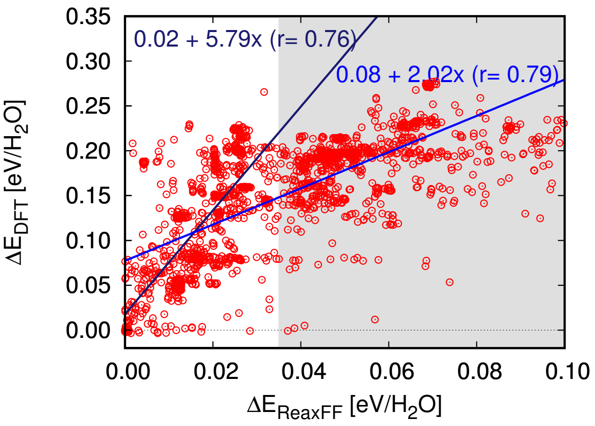

Supplementary Figure 10. Correlation between ReaxFF, $\Delta E_{\rm ReaxFF}$, and PBE-DFT energies, $\Delta E_{\rm DFT}$, measured with respect to Ih. The grey area roughly indicates structures eliminated in the selection cut indicated in Supplementary Fig. 3. For the survivors $\Delta E_{\rm DFT} \sim 5\Delta E_{\rm ReaxFF}$, so that the seemingly tight selection cut in Supplementary Fig. 3 retains (almost) all structures with DFT energies of up to 0.1 eV/H_2O above the CH.

Supplementary Note 6. CHOICE OF *xc*-FUNCTIONAL

Since energy differences of a few meV/H_2O are crucial, for example in determining the relative stabilities of Ih, 0, and XVIII, it is mandatory to investigate the choice of *xc*-functional. Santra *et al.* [16] and Gillan *et al.* [17–19] showed that dispersion-corrected semi-local and hybrid functionals provide a substantially better approximation to the static lattice energies of a wide range of ice phases than uncorrected functionals, when compared to accurate diffusion Monte Carlo and quantum chemical results. The improvement is largest for high density phases. This is reflected in Supplementary Fig. 11 in which the differences between the $E_{\rm st}$ obtained from

	Exp		TIP4P/2005		ReaxFF	PBE-DFT
Ih	0.000	(0.000)	0.000	(0.000)	0.000	0.000
II	0.004	(-0.001)	0.055	(0.055)	0.049	0.065
III	0.056	(0.059)	0.083	(0.084)	NA	0.060
IX	0.021	(0.018)	NA	NA	0.017	0.054
V	0.059	(0.060)	0.108	(0.111)	0.057	0.079
VI	0.101	(0.091)	0.142	(0.143)	0.071	0.109
XII	NA	(0.079)	NA	(0.131)	0.059	0.089
VII	0.265	(0.169)	NA	NA	NA	0.204
VIII	0.197	(0.143)	NA	(0.524)	0.196	0.185

Supplementary Table 5. Differences in energy at 0 K (150 K) and pressure between different known phases of ice, according to the calorimetry experiments of Whalley [9], TIP4P/2005 simulations of Aragones *et al.* [15], and ReaxFF calculations. All energies are measured in eV/H_2O and with respect to ice Ih.

	Exp	TIP4P/2004	ReaxFF
Ih	0.92	0.95	0.96
II	1.17	1.23	1.12
III	1.14	1.18	1.05
V	1.23	1.30	1.19
VI	1.31	1.39	1.30
XII	1.29	NA	1.25
VIII	1.46	NA	1.48

Supplementary Table 6. Comparison of experimental, TIP4P/2005, and ReaxFF ice densities. Experimental densities of different known phases of ice [9] and densities obtained using the TIP4P/2005 [15] and ReaxFF force fields. All densities are measured in g/cm^3.

the PBE and dispersion-corrected PBE-TS [20] functionals are much smaller for the low-density i, 0, and XVII phases of ice and the proposed structure [10], than for the high-density ice phases.

The dependence of $E_{\rm st}$ on the choice of functional arises largely from the changes in static lattice volumes. This begs the question of how accurate PBE and PBE-TS volumes are compared to experiment. The known ice phases provide a reference. In particular, the recently identified ice XVII provides a valuable reference at low density. Analogously to ice Ih, the calculated PBE-DFT density exceeds the experimental density of XVII at 20 K of 0.949 g/cm^3 by 4%. At the same time the calculated and experimental densities of the high-density ice phases agree to within 5%, suggesting that non-dispersion corrected PBE-DFT provides reasonable volumes across all densities.

Moreover, applying the Tkatchenko-Scheffler (TS) dispersion correction [20] to the PBE functional largely retains the energetic ordering of phases of the PBE functional. Nonetheless, a dispersion correction is required for $E_{\rm st}$ to be accurate to within a few meV/H_2O, which

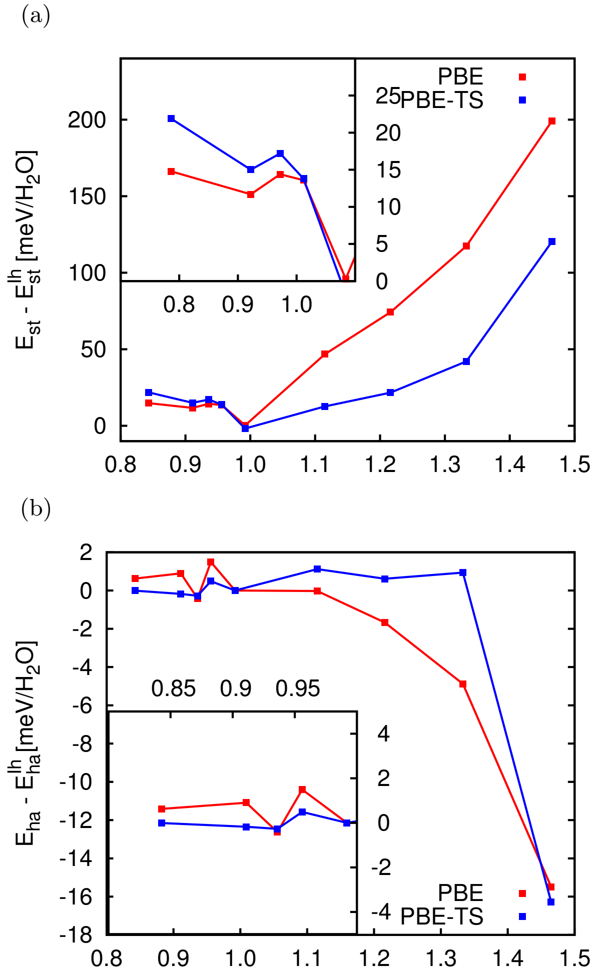

Supplementary Figure 11. Effect of dispersion corrections on configurational energies. (a) PBE and PBE-TS static lattice, E_{st}, and (b) harmonic vibrational energies, E_{ha}, with respect to Ih at zero pressure and temperature for different ice phases ordered by density.

	Relaxed volumes		DMC volumes	
	$E^{\mathrm{i}}_{\mathrm{st}}$	E^{0}_{st}	$E^{\mathrm{i}}_{\mathrm{st}}$	E^{0}_{st}
PBE	14.1	13.3	13.8	13.0
PBE-TS	19.2	15.8	18.3	14.8
DMC			24 ± 5	17 ± 5

Supplementary Table 7. Comparison of DFT and DMC static lattice energies. Static lattice energies with respect to Ih, ΔE_{st}, for ice i and 0 in meV/H_2O obtained at the PBE and PBE-TS relaxed volumes and at the volumes used in the diffusion Monte Carlo (DMC) study of Quigley *et al.* [21]. The DMC results of Quigley *et al.* are shown for comparison. The volumes used for the DMC calculations are 4% larger than the PBE values.

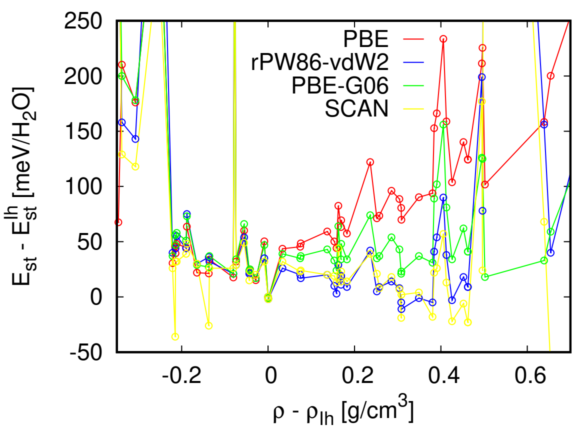

Supplementary Figure 12. E_{st} with respect to ice Ih in meV/H_2O as a function of ρ in g/cm^3 for the 50 synthesisable PBE-relaxed structures highlighted in the main text (and 7 additional structures just more than 20 meV/H_2O above the GCH) obtained using different *xc*-functionals. The E_{st} of the PBE-relaxed structures were calculated using the PBE [26] functional, the PBE functional with the Grimme (G06) dispersion correction [27], the rPW86-vdW2 functional [22], and the SCAN functional [23].

is demonstrated by comparing the E_{st} for ice 0 and i using PBE and PBE-TS with the diffusion Monte Carlo results of Quigley *et al.* [21] (see Supplementary Table 7).

The use of the PBE *xc*-functional can be further justified by investigating how the relative stability of the known and novel structures proposed for synthesis in the main text is affected by the choice of *xc*-functional. Santra *et al.* [16] have shown that the rPW86-vdW2 *xc*-functional [22] produces particularly accurate relative stabilities for the known phases of ice compared to different semi-local *xc*-functionals. More recently the SCAN functional of Sun *et al.* [23] has been shown to reproduce said relative stabilities in equivalently good agreement with experiment [24]. The PBE, rPW86-vdW2, and SCAN energies of the 50 synthesisable PBE-relaxed structures highlighted in the main text and 7 additional structures just more than 20 meV/H_2O above the GCH (calculated using Quantum Espresso [25] with a plane-wave energy cut-off of 40 Rydberg, the same k-point grids employed in the original PBE-DFT calculations, and the O.pbe-rrjkus.UPF, H.pbe-rrjkus.UPF, O.pbe-hgh.UPF, and H.pbe-hgh.UPF pseudopotentials from http://www.quantum-espresso.org.) is shown as a function of density in Supplementary Fig. 12. However, the more stringent and representative test in the context of this work is the comparison of relative stabilities after geometry optimisation with the respective *xc*-functional (see Supplementary Fig. 13). Both figures highlight

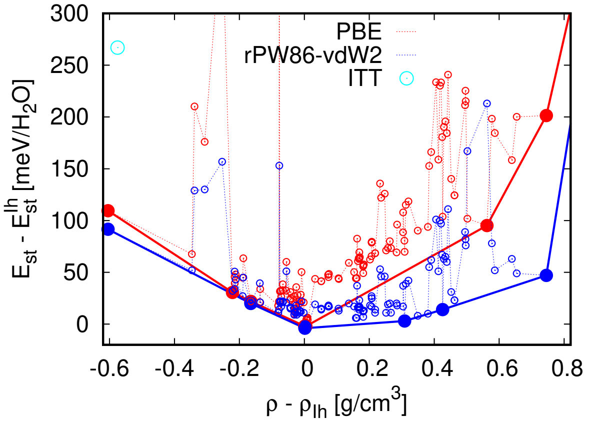

Supplementary Figure 13. $E_{\mathrm{st}}(\rho)$ with respect to ice Ih in meV/H_2O for the 136 structures within 20 meV/H_2O of the original GCH after full geometry-optimisation using the PBE and rPW86-vdW2 functionals, respectively. The respective energy-density CH are shown as solid lines. The CH vertices are highlighted as thick, filled circles. The ice counterpart of the ITT zeolite network, which was suggested as the most stable "aeroice" structure below around -0.4 GPa in Ref. [10], is highlighted in cyan, but is unstable at the PBE level of theory and still far from stable at the rPW86-vdW2 level of theory.

the importance of dispersion effects on the static lattice energies of different ice structures and show that the energy-density CH (only explicitly shown in Supplementary Fig. 13) is sensitive to the choice of xc-functional, even though the set of structures within $\sim 10\,\mathrm{meV}/\mathrm{H_2O}$ is insensitive to the choice of xc-functional. Crucially, the GCH construction described in the main text is largely unaffected by the choice of xc-functional, rendering the central features of the configuration space map shown in Fig. 3 of the main text insensitive to the choice of xc-functional. This is most strikingly demonstrated by re-evaluating the GCH constructed on the first three KPCA descriptors for the 136 most promising candidates in direct analogy with the main text, but using the E_{st} obtained using the rPW86-vdW2 [22] xc-functional. Out of 38 vertices obtained using PBE all but one are recovered using rPW86-vdW2 and only three additional vertices emerge.

Furthermore, harmonic vibrational energies are only weakly affected by the Tkatchenko-Scheffler dispersion correction. In particular, the effect is negligible for the low density i, 0, and XVII phases and structure [10] (see Supplementary Fig. 11).

Anharmonic vibrational motion leads to expansions of the ice structures of a few percent of their static volumes. For example, in XI the zero-point expansion from first-principles calculations is $4.5 \pm 0.5\%$ [3], which is in good agreement with the value of $\sim 4\%$ obtained in the quasi-

	$V_{\mathrm{st}}^{\mathrm{XI}}$ [$\mathring{A}^3$/H_2O]	V^{XI} [$\mathring{A}^3$/H_2O]
PBE	30.26	31.47
PBE-TS	29.14	30.31
PBE-G06	28.93	30.09
Exp	–	32.10

Supplementary Table 8. Volumes per H_2O without (st) and with zero-point QNE for different xc-functionals. The experimental value was taken from Ref. [30].

	$E_{\mathrm{har}}^{\mathrm{XI}}$	$E_{\mathrm{anh}}^{\mathrm{XI}}$	$E_{\mathrm{vib}}^{\mathrm{XI}}$
V_{PBE}	689.76	6.76	696.52
V_{Exp}	689.18	6.93	696.11

Supplementary Table 9. Harmonic and anharmonic contributions and total vibrational energies in [meV/H_2O] of XI using the PBE functional and PBE relaxed and experimental volumes.

harmonic approximation as well as with the path-integral Monte Carlo results from Refs. [28, 29]. The PBE volume including quantum vibrations of $31.9 \pm 0.3\,\mathring{A}^3$ per molecule is within 1% of the experimental value [30]. PBE with a TS or G06 [27] dispersion correction underestimates the volume per molecule by several percent (see Supplementary Table 8).

Neither the static lattice energies nor the harmonic or the anharmonic vibrational energies depend significantly on whether the calculations are performed using relaxed or experimental volumes (see Supplementary Tables 7 and 9). This also implies that the role of thermal expansion of up to 2% close to room temperature for vibrational energies is negligible. This agrees with the conclusion that the vibrational energies are insensitive to the choice of xc-functional and the corresponding choice of (relaxed) volumes. In view of the better agreement with experiment of the PBE volumes without dispersion correction (once the ZP expansion of the unit cell is accounted for), we choose to use the PBE functional without dispersion correction for vibrational calculations.

We do not perform calculations at zero-point expanded volumes, since no experimental data are available for the ice i and 0 phases, or in fact for various structures derived from hypothetical zeolites. Simulations of the zero-point expansion go beyond the scope of this study.

[1] G. A. Tribello, B. Slater, M. A. Zwijnenburg, and R. G. Bell, Isomorphism between ice and silica, *Physical Chemistry Chemical Physics* **12**, 8597 (2010).

[2] S. J. Singer, J.-L. Kuo, T. K. Hirsch, C. Knight, L. Ojamäe, and M. L. Klein, Hydrogen-Bond Topology and the Ice VII/VIII and Ice Ih/XI Proton-Ordering Phase Transitions, *Physical Review Letters* **94**, 135701 (2005).

[3] E. A. Engel, B. Monserrat, and R. J. Needs, Anharmonic nuclear motion and the relative stability of hexagonal and cubic ice, *Phys. Rev. X* **5**, 021033 (2015).

[4] M. M. J. Treacy, K. H. Randall, S. Rao, J. A. Perry, and D. J. Z. Chadi, Enumeration of periodic tetrahedral frameworks, *Zeitschrift für Kristallographie* **212**, 728 (1997).

[5] O. D. Friedrichs, A. W. M. Dress, D. H. Huson, J. Klinowski, and A. L. Mackay, Systematic enumeration of crystalline networks, *Nature* **400**, 644 (1999).

[6] M. M. J. Treacy, I. Rivin, E. Balkovsky, K. H. Randall, and M. D. Foster, Enumeration of periodic tetrahedral frameworks. II. Polynodal graphs, *Microporous Mesoporous Materials* **74**, 121 (2004).

[7] D. J. Earl and M. W. Deem, Toward a Database of Hypothetical Zeolite Structures, *Ind. Eng. Chem. Res.* **45**, 5449 (2006).

[8] C. Baerlocher, W. M. Meier, and D. H. Olson, *Atlas of Zeolite Framework Types*, Elsevier, Amsterdam, 2007.

[9] E. Whalley, Energies of the phases of ice at zero temperature and pressure, *Journal of Chemical Physics* **81**, 4087 (1984).

[10] T. Matsui, M. Hirata, T. Yagasaki, M. Matsumoto, and H. Tanaka, Hypothetical ultralow-density ice polymorphs, *Journal of Chemical Physics* **147**, 091101 (2017).

[11] A. C. T. van Duin, S. Dasgupta, F. Lorant, and W. A. G. III, ReaxFF: A Reactive Force Field for Hydrocarbons, *Journal of Physical Chemistry A* **105**, 9396 (2001).

[12] J. D. Bernal and R. H. Fowler, A Theory of Water and Ionic Solution, with Particular Reference to Hydrogen and Hydroxyl Ions, *Journal of Chemical Physics* **1**, 515 (1933).

[13] J. L. F. Abascal and C. Vega, A general purpose model for the condensed phases of water: TIP4P/2005, *Journal of Chemical Physics* , 234505 (2005).

[14] D. Raymand et al., Water adsorption on stepped ZnO surfaces from MD simulation, *Surface Science* **604**, 741 (2010).

[15] J. L. Aragones, E. G. Noya, J. L. F. Abascal, and C. Vega, Properties of ices at 0 K: A test of water models, *Journal of Chemical Physics* **127**, 154518 (2007).

[16] B. Santra, J. Klimeš, A. Tkatchenko, D. Alfè, B. Slater, A. Michaelides, R. Car, and M. Scheffler, On the Accuracy of van der Waals Inclusive Density-Functional Theory Exchange-Correlation Functionals for Ice at Ambient and High Pressures, *Journal of Chemical Physics* **139**, 154702 (2013).

[17] A. P. Bartók, M. J. Gillan, F. R. Manby, and G. Csányi, Machine-learning approach for one- and two-body corrections to density functional theory: Applications to molecular and condensed water, *Physical Review B* **88**, 054104 (2013).

[18] M. J. Gillan, D. Alfé, A. P. Bartók, and G. Csányi, First-principles energetics of water clusters and ice: A many-body analysis, *Journal of Chemical Physics* **139**, 244504 (2013).

[19] M. J. Gillan, F. R. Manby, M. D. Towler, and D. Alfé, Assessing the accuracy of quantum Monte Carlo and density functional theory for energetics of small water clusters, *Journal of Chemical Physics* **136**, 244105 (2012).

[20] A. Tkatchenko and M. Scheffler, Accurate Molecular Van Der Waals Interactions from Ground-State Electron Density and Free-Atom Reference Data, *Physical Review Letters* **102**, 073005 (2009).

[21] D. Quigley, D. Alfé, and B. Slater, Communication: On the stability of ice 0, ice i, and Ih, *Journal of Chemical Physics* **141**, 161102 (2014).

[22] K. Lee, E. D. Murray, L. Kong, B. I. Lundqvist, and D. C. Langreth, Higher-accuracy van der Waals density functional, *Physical Review B* **82**, 081101(R) (2010).

[23] J. Sun, A. Ruzsinszky, and J. P. Perdew, Strongly Constrained and Appropriately Normed Semilocal Density Functional, *Physical Review Letters* **115**, 036402 (2015).

[24] J. Sun, R. C. Remsing, Y. Zhang, Z. Sun, A. Ruzsinszky, H. Peng, Z. Yang, A. Paul, U. Waghmare, X. Wu, M. L. Klein, and J. P. Perdew, Accurate first-principles structures and energies of diversely bonded systems from an efficient density functional, *Nature Chemistry* **8**, 831 (2016).

[25] P. Giannozzi, S. Baroni, N. Bonini, M. Calandra, R. Car, C. Cavazzoni, D. Ceresoli, G. L. Chiarotti, M. Cococcioni, I. Dabo, A. Dal Corso, S. Fabris, G. Fratesi, S. de Gironcoli, R. Gebauer, U. Gerstmann, C. Gougoussis, A. Kokalj, M. Lazzeri, L. Martin-Samos, N. Marzari, F. Mauri, R. Mazzarello, S. Paolini, A. Pasquarello, L. Paulatto, C. Sbraccia, S. Scandolo, G. Sclauzero, A. P. Seitsonen, A. Smogunov, P. Umari, and R. M. Wentzcovitch, QUANTUM ESPRESSO: a modular and open-source software project for quantum simulations of materials, *Journal of Physics: Condensed Matter* **21**, 395502 (2009).

[26] J. P. Perdew, K. Burke, and M. Ernzerhof, Generalized Gradient Approximation Made Simple, *Physical Review Letters* **77**, 3865 (1996).

[27] S. Grimme, Semiempirical GGA-type density functional constructed with a long-range dispersion correction, *Journal of Computational Chemistry* **27**, 1787 (2006).

[28] R. Ramírez, N. Neuerburg, M.-V. Fernández-Serra, and C. P. Herrero, Quasi-harmonic approximation of thermodynamic properties of ice Ih, II, and III, *Journal of Chemical Physics* **137**, 044502 (2012).

[29] C. P. Herrero and R. Ramírez, High-density amorphous ice: A path-integral simulation, *Journal of Chemical Physics* **137**, 104505 (2012).

[30] L. G. Dowell and A. P. Rinfret, Low-temperature Forms of Ice as studied by X-ray Diffraction, *Nature* **188**, 1144 (1960).
