# Supplementary material for: Mapping uncharted territory in ice from zeolite networks to ice structures
Source: Nat Commun. 2018 Jun 5;9:2173. doi: 10.1038/s41467-018-04618-6 (PMC5988809; doi:10.1038/s41467-018-04618-6)
Supplement: Supplementary file 2 — Supplementary Data 1 [file 41467_2018_4618_MOESM2_ESM.pdf]

Supplementary Material: Mapping uncharted territory in ice

Edgar A. Engel and Richard J. Needs

TCM Group, Cavendish Laboratory, J J Thomson Avenue, Cambridge, CB3 0HE, UK

Chris J. Pickard

Department of Materials Science and Metallurgy,

27 Charles Babbage Road, Cambridge CB3 0FS, UK and

Advanced Institute for Materials Research, Tohoku University,

2-1-1 Katahira, Aoba, Sendai 980-8577, Japan

Andrea Anelli and Michele Ceriotti

Laboratory of Computational Science and Modeling, Institute of Materials,

École Polytechnique Fédérale de Lausanne, 1015 Lausanne, Switzerland

(Dated: April 18, 2018)

Abstract

This supplementary information provides the structural information for the 18 new candidate ice structures highlighted in the main text in terms of CASTEP .cell-files. Sample CASTEP input files for a geometry optimisation of one particular structure specify the computational details of the geometry optimisations used throughout.

STRUCTURAL DATA

BSV

```
%BLOCK lattice_cart
12.870585  -0.013532  -0.022867
 -4.302907  12.129375  -0.022593
 -4.302439  -6.083988  10.491005
%ENDBLOCK lattice_cart
%BLOCK positions_frac
H 0.080245 0.456467 0.736819
H 0.919769 0.543549 0.263170
H 0.279895 0.042826 0.122846
H 0.720072 0.957162 0.877154
H 0.418961 0.156482 0.376384
H 0.581048 0.843510 0.623623
H 0.219899 0.343048 0.763280
H 0.780136 0.656951 0.236732
H 0.043562 0.263182 0.419791
H 0.956469 0.736811 0.580240
H 0.236814 0.580221 0.456481
H 0.763191 0.419777 0.543486
H 0.156964 0.236728 0.280139
H 0.843071 0.763279 0.719893
H 0.622895 0.779959 0.042919
H 0.377115 0.220060 0.957092
H 0.542809 0.122819 0.779876
H 0.457159 0.877193 0.220098
H 0.123619 0.081036 0.843485
H 0.876341 0.918963 0.156526
H 0.343466 0.623619 0.081023
H 0.656512 0.376388 0.918948
H 0.263261 0.719867 0.343076
H 0.736734 0.280107 0.656924
H 0.263157 0.043555 0.919780
H 0.736835 0.956475 0.080239
H 0.122836 0.542858 0.279927
H 0.877165 0.457124 0.720058
H 0.376388 0.656529 0.418990
H 0.623637 0.343474 0.581005
H 0.236727 0.156934 0.780130
H 0.763273 0.843057 0.219888
H 0.580195 0.236811 0.956431
H 0.456440 0.080216 0.236807
H 0.543541 0.919770 0.763185
H 0.280113 0.736749 0.156941
H 0.719855 0.263240 0.843042
H 0.042848 0.279904 0.622823
H 0.957151 0.720078 0.377153
H 0.220083 0.377120 0.457128
H 0.779968 0.622890 0.542904
H 0.156538 0.418975 0.876417
H 0.843497 0.581064 0.123606
H 0.081020 0.123617 0.343503
H 0.918987 0.876361 0.656530
H 0.343008 0.219838 0.263272
H 0.656965 0.780161 0.736734
H 0.081102 0.392119 0.538828
H 0.918917 0.607881 0.461181
H 0.146667 0.107710 0.188522
H 0.853326 0.892283 0.811493
H 0.581385 0.042342 0.689525
H 0.418607 0.957669 0.310466
H 0.646903 0.457699 0.037979
H 0.353091 0.542307 0.962006
H 0.107869 0.461203 0.418921
H 0.892157 0.538789 0.581068
H 0.038797 0.581069 0.392106
H 0.961232 0.418973 0.607876
H 0.957683 0.037981 0.146905
H 0.042300 0.962013 0.853113
H 0.688574 0.646717 0.107732
H 0.311500 0.353307 0.892275
H 0.607730 0.188533 0.646679
H 0.392240 0.811449 0.353305
H 0.189503 0.081368 0.042334
H 0.810442 0.918608 0.957660
H 0.542335 0.689561 0.081423
H 0.457667 0.310464 0.918573
H 0.538014 0.146913 0.457703
H 0.461990 0.853071 0.542326
H 0.461178 0.107848 0.918887
H 0.538791 0.892176 0.081075
H 0.188508 0.607730 0.146676
H 0.811435 0.392253 0.853296
H 0.310480 0.457700 0.418627
H 0.689578 0.542319 0.581402
H 0.962005 0.042301 0.353124
H 0.038003 0.957654 0.646908
H 0.418928 0.961178 0.107865
H 0.581046 0.038817 0.892104
H 0.392089 0.081055 0.038780
H 0.607891 0.918932 0.961216
H 0.853079 0.462007 0.042318
H 0.146923 0.538009 0.957706
H 0.107749 0.146699 0.688540
H 0.892263 0.853285 0.311459
H 0.353293 0.311452 0.392286
H 0.646723 0.688564 0.607738
H 0.042352 0.581426 0.189542
H 0.957678 0.418602 0.810467
H 0.081372 0.189503 0.542322
H 0.918612 0.810458 0.457666
H 0.542307 0.353100 0.461985
H 0.457694 0.646907 0.538024
O 0.041586 0.414766 0.642063
O 0.958407 0.585207 0.357928
O 0.227268 0.084554 0.126484
O 0.772750 0.915483 0.873542
O 0.457309 0.100484 0.372763
O 0.542719 0.899533 0.627234
O 0.726911 0.600628 0.141862
O 0.273099 0.399365 0.858143
O 0.085100 0.357913 0.458335
O 0.914885 0.642078 0.541657
O 0.142080 0.541615 0.414810
O 0.857926 0.458356 0.585134
O 0.100615 0.141876 0.226892
O 0.899368 0.858132 0.773088
O 0.626490 0.727314 0.084641
O 0.373532 0.272727 0.915390
O 0.584623 0.126498 0.727311
O 0.415404 0.873529 0.272720
O 0.127220 0.042706 0.899529
O 0.872734 0.957322 0.100512
O 0.399508 0.627232 0.042702
O 0.600481 0.372774 0.957277
O 0.641884 0.226854 0.600581
O 0.358111 0.773071 0.399385
O 0.357882 0.085125 0.958330
O 0.642104 0.914900 0.041682
O 0.126457 0.584557 0.227252
O 0.873530 0.415391 0.772695
O 0.372784 0.600486 0.457315
O 0.627252 0.399490 0.542656
O 0.141873 0.100630 0.726932
O 0.858129 0.899388 0.273139
O 0.458410 0.857943 0.085228
O 0.541567 0.142065 0.914752
O 0.414841 0.041608 0.142054
O 0.585164 0.958401 0.857938
O 0.226899 0.641896 0.100622
O 0.773106 0.358098 0.899384
O 0.084595 0.227284 0.626482
O 0.915371 0.772666 0.373495
O 0.272718 0.373532 0.415400
O 0.727288 0.626465 0.584591
O 0.100487 0.457273 0.872805
O 0.899517 0.542734 0.127228
O 0.042680 0.127238 0.399522
O 0.957296 0.872752 0.600475
O 0.399365 0.273109 0.358133
O 0.600660 0.726943 0.641871
%ENDBLOCK positions_frac
```

DDR

```
%BLOCK lattice_cart
10.943623  0.369952   0.177150
  5.790014  9.292627   0.043152
  5.750655  3.173819  12.142493
%ENDBLOCK lattice_cart
%BLOCK positions_frac
H   0.333801 0.602935   0.248604
H   0.666195 0.397068   0.751385
H   0.198237 0.166515   0.749722
H   0.801765 0.833478   0.250279
H   0.142142 0.311827   0.229646
H   0.857851 0.688185   0.770353
H   0.201038 0.384438   0.743284
H   0.798979 0.615554   0.256703
H   0.143576 0.805395   0.226213
H   0.856408 0.194594   0.773793
H   0.326683 0.296082   0.258086
H   0.673309 0.703937   0.741902
H   0.548847 0.521042   0.234963
H   0.451148 0.478966   0.765048
H   0.721111 0.007173   0.252592
H   0.278902 0.992824   0.747403
H   1.012551 0.215060   0.258592
H  -0.012550 0.784946   0.741400
H   0.685647 0.534144   0.242452
H   0.314345 0.465878   0.757546
H   1.019237 0.041642   0.235489
H  -0.019247 0.958349   0.764525
H   0.550947 0.203708   0.227883
H   0.449053 0.796290   0.772115
H   0.402960 0.419894   0.290976
H   0.597043 0.580111   0.709007
H   0.087326 0.100368   0.697375
H   0.912678 0.899621   0.302608
H   0.087304 0.626288   0.699896
H   0.912695 0.373726   0.300089
H   0.315954 0.499127   0.421770
H   0.684044 0.500884   0.578211
H   0.255623 0.174312   0.576095
H   0.744391 0.825689   0.423895
H   0.011191 0.237715   0.424712
H   0.988834 0.762309   0.575258
H   0.261740 0.462806   0.613033
H   0.738276 0.537193   0.386959
H   0.007874 0.850669   0.387144
H   0.992128 0.149334   0.612842
H   0.349210 0.241187   0.382480
H   0.650798 0.758821   0.617507
H   0.280504 0.311883   0.111259
H   0.719486 0.688141   0.888736
H   0.281597 0.793168   0.105424
H   0.718387 0.206820   0.894577
H   0.185502 0.214497   0.896273
H   0.814495 0.785511   0.103730
H   0.178248 0.510614   0.143819
H   0.821735 0.489389   0.856191
H   0.178543 0.665887   0.137455
H   0.821447 0.334109   0.862563
H   0.032470 0.309405   0.853376
H   0.967521 0.690592   0.146637
H   0.638593 0.649619   0.078091
H   0.361403 0.350379   0.921922
H   0.644496 0.142059   0.073605
H   0.355504 0.857928   0.926410
H   0.161120 0.133234   0.076179
H   0.838883 0.866755   0.923823
H   0.477759 0.215130  -0.001353
H   0.522228 0.784872   0.001384
H   0.680490 0.023554   0.007129
H   0.319500 0.976433  -0.007106
H   0.299825 0.183004   0.001568
H   0.700168 0.816993  -0.001540
H   0.743624 0.398557   0.134832
H   0.256374 0.601425   0.865192
H   0.709891 0.219971   0.147853
H   0.290125 0.780006   0.852165
H   0.938762 0.201576   0.150234
H   0.061236 0.798408   0.849778
H   0.232557 0.396136   0.502870
H   0.767458 0.603867   0.497112
H   0.144885 0.255480   0.496641
H   0.855130 0.744514   0.503345
H   0.094746 0.640936   0.500347
H   0.905275 0.359073   0.499631
H   0.178326 0.660493  -0.018182
H   0.821665 0.339502   1.018200
O   0.402988 0.510013   0.295615
O   0.597006 0.489994   0.704377
O   0.227194 0.099754   0.701936
O   0.772825 0.900240   0.298057
O   0.009540 0.273949   0.301452
O   0.990462 0.726066   0.698533
O   0.233914 0.466437   0.692069
O   0.766091 0.533564   0.307922
O   0.002900 0.900000   0.307353
O   0.997101 0.099998   0.692631
O   0.394848 0.275639   0.300741
O   0.605151 0.724376   0.699243
O   0.229676 0.340219   0.185695
O   0.770315 0.659795   0.814307
O   0.232886 0.735190   0.176617
O   0.767092 0.264799   0.823387
O   0.148554 0.265645   0.826737
O   0.851437 0.734355   0.173273
O   0.634150 0.726192   0.003659
O   0.365841 0.273813   0.996359
O   0.637585 0.135118   0.004769
O   0.362404 0.864871   0.995252
O   0.241626 0.134653   0.002192
O   0.758383 0.865336   0.997820
O   0.646913 0.531281   0.189005
O   0.353084 0.468721   0.811001
O   0.655019 0.156398   0.174072
O   0.344987 0.843588   0.825935
O   0.030031 0.135705   0.183131
O   0.969967 0.864283   0.816880
O   0.244921 0.483406   0.498309
O   0.755098 0.516595   0.501677
O   0.239293 0.245643   0.501833
O   0.760714 0.754362   0.498157
O  -0.002463 0.256115   0.495465
O   0.002479 0.743890   0.504514
O   0.802838 0.305977   0.102083
O   0.197151 0.694012   0.897936
O   0.141548 0.623543   0.108835
O   0.858451 0.376456   0.891168
%ENDBLOCK positions_frac
```

LTA

```
%BLOCK lattice_cart
 9.164225 -0.000015 -0.000014
-0.000015  9.164213  0.000021
-0.000014  0.000021  9.164197
%ENDBLOCK lattice_cart
%BLOCK positions_frac
H -0.000007  0.206477  0.500097
H  0.000000  0.793503  0.499981
H  0.499992  0.000000  0.206488
H  0.500035 -0.000008  0.793517
H  0.206492  0.500159 -0.000004
H  0.793516  0.499836 -0.000006
H  0.206496 -0.000001  0.500039
H  0.793492 -0.000010  0.499942
H  0.000002  0.500215  0.793489
H  0.000001  0.499932  0.206491
H  0.499839  0.206495 -0.000003
H  0.500148  0.793490  0.000006
H  0.101133  0.101130  0.359159
H  0.898868  0.898848  0.640836
H  0.898834  0.898892  0.359189
H  0.101114  0.101136  0.640797
H  0.898877  0.101143  0.640815
H  0.101133  0.898860  0.359173
H  0.101146  0.898879  0.640818
H  0.898932  0.101194  0.359174
H  0.359219  0.101120  0.101115
H  0.640783  0.898906  0.898854
H  0.359140  0.898895  0.898807
H  0.640860  0.101127  0.101166
H  0.640797  0.898850  0.101107
H  0.359206  0.101170  0.898924
H  0.640846  0.101127  0.898850
H  0.359152  0.898881  0.101171
H  0.101147  0.359150  0.101148
H  0.898791  0.640832  0.898910
H  0.898885  0.359187  0.898871
H  0.101097  0.640791  0.101136
H  0.101209  0.640767  0.898951
H  0.898839  0.359218  0.101083
H  0.898903  0.640856  0.101176
H  0.101096  0.359120  0.898802
H  0.000001  0.282297  0.282312
H -0.000011  0.717674  0.717651
H -0.000002  0.717703  0.282313
H  0.000008  0.282379  0.717787
H  0.282280 -0.000005  0.282334
H  0.717748  0.000001  0.717635
H  0.282337 -0.000018  0.717715
H  0.717675 -0.000003  0.282309
H  0.282165  0.282459 -0.000000
H  0.717780  0.717600 -0.000005
H  0.717555  0.282177  0.000004
H  0.282472  0.717843 -0.000009
O -0.000026  0.186204  0.369542
O -0.000013  0.813770  0.630427
O  0.000018  0.813768  0.369604
O -0.000003  0.186207  0.630353
O  0.369604 -0.000019  0.186213
O  0.630382  0.000004  0.813798
O  0.369566 -0.000040  0.813771
O  0.630422  0.000026  0.186228
O  0.186265  0.369489  0.000017
O  0.813766  0.630509 -0.000036
O  0.813810  0.369699  0.000013
O  0.186179  0.630304 -0.000033
O  0.186223 -0.000011  0.630387
O  0.813762 -0.000037  0.369622
O  0.813755 -0.000003  0.630443
O  0.186233  0.000008  0.369570
O  0.000027  0.369503  0.813787
O -0.000020  0.630434  0.186207
O  0.000026  0.369623  0.186234
O -0.000017  0.630325  0.813725
O  0.369699  0.186191 -0.000019
O  0.630315  0.813786  0.000023
O  0.369493  0.813724  0.000006
O  0.630516  0.186262 -0.000001
%ENDBLOCK positions_frac
```

NON

```
%BLOCK lattice_cart
 8.412896  0.109847 -0.029323
-0.865616  8.408499 -0.036097
-3.806821 -4.303925  8.879616
%ENDBLOCK lattice_cart
%BLOCK positions_frac
H 0.531363 0.667298 0.193653
H 0.508811 0.308772 0.793399
H 0.020181 0.163091 0.181444
H 0.970542 0.837664 0.809695
H 0.216268 0.024181 0.206934
H 0.836572 0.969386 0.807955
H 0.663711 0.533001 0.192859
H 0.336535 0.476666 0.817016
H 0.540583 0.268547 0.600638
H 0.454437 0.731099 0.399164
H 0.121231 0.412034 0.353458
H 0.879052 0.588097 0.645560
H 0.408797 0.121005 0.355777
H 0.579923 0.878612 0.646216
H 0.268340 0.531874 0.599272
H 0.731300 0.467642 0.400617
H 0.368565 0.847419 0.246388
H 0.611168 0.147717 0.753792
H 0.846459 0.376447 0.245545
H 0.150910 0.620534 0.753900
H 0.277387 0.632748 0.180847
H 0.761341 0.418293 0.842860
H 0.056041 0.406741 0.184453
H 0.937249 0.591744 0.811908
H 0.420077 0.080656 0.157878
H 0.593904 0.942548 0.817188
H 0.631880 0.281042 0.186998
H 0.364925 0.718289 0.815840
H 0.502461 -0.023533 -0.013902
H 0.016043 0.490633 0.013149
H 0.365684 0.162218 0.033823
H 0.627099 0.838571 0.971544
H 0.156893 0.368763 0.027665
H 0.829846 0.627198 0.966238
H 0.406774 0.635059 0.496320
H 0.594261 0.364828 0.504461
H 0.358304 0.399754 0.503989
H 0.642130 0.601128 0.496104
H 0.110061 0.110072 0.875311
H 0.883329 0.888710 0.120122
H 0.263293 0.267106 0.879432
H 0.734981 0.735861 0.125113
H 0.001310 0.007764 0.025490
H 0.475846 0.481333 -0.026204
O 0.392153 0.730917 0.247728
O 0.596220 0.266156 0.751117
O 0.019286 0.355796 0.246629
O 0.979430 0.643650 0.753190
O 0.345655 0.020105 0.248510
O 0.642292 0.979064 0.753616
O 0.729007 0.396115 0.248620
O 0.268908 0.601675 0.751094
O 0.465407 0.123725 0.095649
O 0.527645 0.877324 0.909920
O 0.118586 0.469037 0.089179
O 0.867860 0.527118 0.903929
O 0.282696 0.486303 0.500494
O 0.717073 0.513884 0.499494
O 0.496615 0.283054 0.502381
O 0.503665 0.717193 0.497898
O 0.026351 0.035063 0.134120
O 0.964954 0.965266 0.854290
O 0.611980 0.614060 0.147416
O 0.392579 0.397902 0.864782
O 0.216352 0.217226 0.935965
O 0.779419 0.780757 0.064067
%ENDBLOCK positions_frac
```

SGT

```
%BLOCK lattice_cart
 8.380192 -0.035881 -0.096055
-0.037150  8.287394 -0.055025
-4.324093 -4.210500 14.262907
%ENDBLOCK lattice_cart
%BLOCK positions_frac
H  0.099112  0.068587 0.200732
H  0.901459  0.931021 0.799018
H  0.081141  0.635899 0.173260
H  0.919979  0.364785 0.827050
H  0.432187  0.402347 0.295332
H  0.567677  0.598013 0.704886
H  0.137387  0.600066 0.701233
H  0.862737  0.399797 0.298569
H  0.978630  0.277819 0.246298
H  0.022178  0.722158 0.753787
H  0.218817  0.520300 0.256239
H  0.781519  0.480051 0.743834
H -0.029012  0.484024 0.742352
H  1.029443  0.516538 0.257702
H  0.219524  0.241508 0.252373
H  0.780987  0.758134 0.747609
H  0.175506  0.131415 0.355741
H  0.824314  0.868001 0.644016
H  0.182008  0.735345 0.363637
H  0.817671  0.264533 0.636426
H  0.366047  0.346189 0.176144
H  0.634277  0.653760 0.824031
H  0.764499  0.317058 0.140870
H  0.236417  0.682749 0.859213
H  0.048248  0.824828 0.106610
H  0.952557  0.175086 0.893325
H  0.276820  0.543983 0.603706
H  0.721933  0.455963 0.395860
H  0.934250 -0.034060 0.067060
H  0.066162  1.033837 0.932812
H  0.179034  0.569467 0.049827
H  0.822030  0.430618 0.950629
H  0.064878  0.411305 0.930600
H  0.936249  0.589008 0.069721
H  0.173097 -0.022719 0.048658
H  0.827060  1.022751 0.951276
H  0.473688  0.436376 0.566251
H  0.525530  0.564150 0.433970
H  0.076062  0.673556 0.549033
H  0.922525  0.326093 0.450377
H  0.095726  0.428121 0.566842
H  0.902898  0.571766 0.432579
H  0.526387  0.366659 0.432873
H  0.472822  0.633837 0.567397
H  0.093551  0.781032 0.477544
H  0.905121  0.218905 0.522039
H  0.387298  0.194940 0.472381
H  0.611673  0.805334 0.527525
H  0.115881  0.166672  0.450998
H  0.883090  0.832822  0.548528
H  0.601465  0.214406  0.525768
H  0.397386  0.785800  0.474236
H  0.724960  0.410941  0.026029
H  0.275920  0.589208  0.974383
H  0.331036  0.173549  0.045162
H  0.669371  0.826147  0.954983
H  0.333794  0.373917  0.043465
H  0.666896  0.625886  0.956854
H  0.725258  0.109109  0.025381
H  0.274982  0.890749  0.974643
H  0.789638  0.061354  0.578150
H  0.209741  0.938544  0.421866
H  0.566078  0.287075  0.075838
H  0.434559  0.712792  0.924495
O  0.132914  0.167342  0.261691
O  0.867664  0.832055  0.738184
O  0.128492  0.598730  0.267008
O  0.872004  0.401839  0.733119
O  0.332591  0.377840  0.236349
O  0.667442  0.622385  0.763746
O  0.104540  0.634516  0.762225
O  0.896436  0.365555  0.237831
O  0.050042  0.947797  0.104614
O  0.950440  0.052096  0.895221
O  0.052910  0.647622  0.106666
O  0.948228  0.352785  0.893589
O  0.546392  0.447626  0.397109
O  0.452836  0.552879  0.603140
O  0.155006  0.544206  0.604621
O  0.843652  0.455659  0.394748
O -0.012824  0.261599  0.493825
O  0.011360  0.738079  0.505598
O  0.497208  0.269474  0.499388
O  0.501722  0.730853  0.500673
O  0.235190  0.504986  0.002905
O  0.765884  0.495114 -0.002451
O  0.232091  0.001355  0.004229
O  0.768101 -0.001449 -0.004328
O  0.204791  0.115731  0.421632
O  0.794529  0.883951  0.578186
O  0.218786  0.819150  0.429083
O  0.780320  0.180787  0.570972
O  0.391628  0.291818  0.077700
O  0.608937  0.707834  0.922543
O  0.687184  0.284301  0.073267
O  0.313345  0.715581  0.926891
%ENDBLOCK positions_frac
```

ACO

```
%BLOCK lattice_cart
 7.557541 -0.000015 0.000005
-0.000015  7.557534 0.000001
 0.000005  0.000001 7.557548
%ENDBLOCK lattice_cart
%BLOCK positions_frac
H  0.249997 0.249500  0.249517
H  0.749987 0.749963  0.749993
H  0.749992 0.750002  0.249526
H  0.249568 0.249546  0.749946
H  0.749958 0.249532  0.749957
H  0.249507 0.749990  0.249989
H  0.249910 0.750082  0.750086
H  0.750046 0.249957  0.249947
H  0.173741 0.173756 -0.000056
H  0.826249 0.826256 -0.000035
H  0.826231 0.173770 -0.000037
H  0.173773 0.826235  0.000046
H  0.000029 0.173774  0.173762
H -0.000048 0.826238  0.826224
H -0.000037 0.826253  0.173747
H -0.000000 0.173752  0.826253
H  0.173753 0.000029  0.173749
H  0.826249 0.000057  0.826247
H  0.173762 0.000046  0.826230
H  0.826232 0.000007  0.173761
H  0.673766 0.673752  0.499955
H  0.326243 0.326219  0.499986
H  0.326253 0.673734  0.500038
H  0.673764 0.326232  0.500010
H  0.499982 0.673731  0.673759
H  0.500016 0.326239  0.326256
H  0.499987 0.326211  0.673776
H  0.499999 0.673755  0.326247
H  0.673773 0.500024  0.673775
H  0.326242 0.500010  0.326246
H  0.673756 0.499918  0.326257
H  0.326254 0.500081  0.673760
O  0.159136 0.159159  0.159178
O  0.840871 0.840833  0.840852
O  0.840849 0.840856  0.159168
O  0.159150 0.159132  0.840864
O  0.840837 0.159148  0.840859
O  0.159168 0.840841  0.159122
O  0.159190 0.840814  0.840801
O  0.840819 0.159176  0.159175
O  0.659183 0.659141  0.659184
O  0.340833 0.340832  0.340861
O  0.340848 0.340789  0.659178
O  0.659158 0.659185  0.340867
O  0.340840 0.659151  0.340817
O  0.659166 0.340821  0.659169
O  0.659150 0.340869  0.340852
O  0.340867 0.659105  0.659134
%ENDBLOCK positions_frac
```

91_2_8335121

```
%BLOCK lattice_cart
 6.331905  0.678210  0.172529
 0.341629  2.362688  0.104548
-0.194688 -0.045858 19.243930
%ENDBLOCK lattice_cart
%BLOCK positions_frac
H  0.463335 -0.088013  0.498720
H -0.607678 -0.046265 -0.000666
H -0.414350  0.226492 -0.258296
H  0.335401  0.644254  0.226276
H  0.532116  0.357109  0.312085
H -0.596368 -0.563738 -0.174365
H -0.557056  0.190576 -0.397621
H  0.485787 -0.188326  0.113706
H  0.541499  0.329638  0.443110
H  0.326523 -0.434255 -0.058098
H -0.366171  0.404981 -0.147177
H  0.256192 -0.144595 -0.395548
H  0.298973  0.482060  0.335119
H -0.348970  0.191628  0.084981
H -0.374800 -0.408705 -0.320721
H  0.280515  0.295615  0.166551
H -0.112527 -0.164334 -0.314721
H -0.013667 -0.035167  0.181288
H -0.192565 -0.297216 -0.492987
H -0.216373  0.834051  0.331830
H  0.077489  0.079555  0.033523
H  0.105006 -0.993987 -0.191991
H -0.314156 -0.064599 -0.047549
H  0.246523 -0.022148  0.428729
H  0.078479 -0.727458 -0.322117
H -0.195626  0.592058  0.191372
H  0.049971 -0.229006  0.457040
H -0.130334  0.326492 -0.055385
H -0.022027 -0.292500 -0.142381
H -0.050294  0.236417  0.352625
H -0.053634  0.574814  0.078603
H -0.048972 -0.406484 -0.429803
O  0.539971 -0.115768  0.452746
O -0.650595 -0.018121 -0.049594
O -0.468972 -0.385463 -0.184161
O -0.626046 -0.063437 -0.424731
O  0.401823  0.207404  0.305102
O  0.570927 -0.111427  0.073114
O -0.382022  0.162040 -0.308987
O  0.279838  0.736876  0.178377
O  0.104891 -0.433818 -0.173447
O -0.181639  0.404043  0.322565
O  0.117547 -0.152965  0.413358
O -0.175868  0.153089  0.177791
O -0.191194  0.009668 -0.073435
O  0.049651 -0.239420 -0.323133
O  0.049165  0.539380  0.040609
O -0.094826 -0.616247 -0.466185
%ENDBLOCK positions_frac
```

53_3_726600

```
%BLOCK lattice_cart
13.982562 -4.048242 -0.028155
 -1.564940  4.777418  0.030278
 -0.001010 -0.031820  4.704863
%ENDBLOCK lattice_cart
%BLOCK positions_frac
H  0.042423  0.343906 -0.078038
H -0.045439 -0.343094  0.066296
H  0.456036 -0.335099  0.425221
H -0.456873  0.346079 -0.434144
H  0.068165 -0.575446  0.427867
H  0.430541  0.583357 -0.069232
H -0.070875  0.574652 -0.439035
H -0.430499 -0.568808  0.061487
H  0.193732  0.395990  0.242848
H -0.196249  0.390693  0.251229
H  0.305005 -0.389956 -0.257341
H -0.194724 -0.390437 -0.252720
H  0.195211 -0.381731 -0.259652
H -0.306944  0.393881  0.249415
H  0.303541  0.387420  0.240698
H -0.305492 -0.387371 -0.250451
H  0.124492 -0.259256  0.242373
H -0.056795 -0.095413  0.245484
H  0.375492  0.267909 -0.255908
H -0.129505  0.257084 -0.254846
H  0.054413  0.096639 -0.254688
H -0.372332 -0.252862  0.248531
H  0.443744 -0.089480  0.246901
H -0.445236  0.099643 -0.254070
H  0.178446  0.078506 -0.083128
H -0.178762 -0.073338  0.414024
H  0.319869 -0.072736 -0.582970
H -0.179960 -0.074877  0.075067
H  0.176695  0.073829 -0.421966
H -0.321327  0.079572  0.578092
H  0.321970 -0.067681  0.078086
H -0.323249  0.076288 -0.083349
O  0.110619 -0.473564  0.245892
O -0.029156 -0.239162  0.256180
O  0.388366  0.480976 -0.251603
O -0.113279  0.474237 -0.256748
O  0.026141  0.238496 -0.267066
O -0.388331 -0.469759  0.244282
O  0.472345 -0.230675  0.235771
O -0.472598  0.243982 -0.243614
O  0.221838  0.253899  0.247924
O -0.278026  0.253723  0.245686
O  0.276909 -0.247843 -0.252345
O -0.223715 -0.250359 -0.256199
O  0.132511 -0.044199 -0.250777
O -0.134232  0.045676  0.243634
O  0.365800  0.050768  0.249701
O -0.367525 -0.041869 -0.254490
%ENDBLOCK positions_frac
```

61_2_8842

```
%BLOCK lattice_cart
7.125918 -0.079424  0.058654
0.034373  7.104955 -0.037821
0.008189 -0.216000  7.058855
%ENDBLOCK lattice_cart
%BLOCK positions_frac
H  0.490373 -0.481889  0.001033
H -0.020047 -0.494042  0.455329
H -0.016925 -0.029336 -0.006382
H -0.493453  0.002728 -0.491846
H -0.450655  0.245616  0.372083
H  0.043185 -0.279410 -0.131604
H  0.501434 -0.202345 -0.400376
H -0.007586 -0.288197  0.371356
H  0.477049  0.223720 -0.129408
H  0.032064  0.226960  0.111915
H  0.031781  0.259405 -0.395212
H  0.478508 -0.276175  0.102866
H  0.220579  0.122789  0.025425
H  0.248926 -0.115874  0.457818
H -0.284492 -0.112226  0.023582
H  0.238543 -0.378316 -0.053735
H  0.283045  0.383772  0.447016
H -0.201408  0.108024 -0.470152
H -0.263580  0.385747 -0.053216
H -0.266270 -0.375387 -0.484683
H  0.388585  0.486251  0.263616
H  0.121139  0.461531 -0.308894
H -0.366354  0.477178 -0.239137
H  0.134002 -0.025394  0.279376
H  0.390407  0.032823 -0.232438
H -0.103533  0.483740  0.190534
H -0.111898 -0.042491 -0.210128
H -0.368529  0.050797  0.268457
H -0.006882 -0.005377 -0.475752
H -0.485903 -0.011007  0.016941
H -0.471631 -0.465572 -0.487410
H -0.000302  0.479192 -0.013008
O  0.109002  0.106928  0.113962
O  0.422775 -0.084517 -0.416623
O -0.097484 -0.116811 -0.090457
O  0.404330 -0.398191  0.083551
O  0.104564  0.377627 -0.426758
O -0.364147  0.134528  0.388068
O -0.383985  0.389896 -0.129171
O -0.095589 -0.400740  0.380522
O  0.406747  0.408518  0.380201
O  0.126478 -0.395123 -0.140986
O -0.384293 -0.385304 -0.402943
O  0.125981 -0.117373  0.385678
O  0.390208  0.112263 -0.109423
O -0.086728  0.401981  0.070029
O -0.090506  0.076408 -0.388842
O -0.404667 -0.090822  0.100596
%ENDBLOCK positions_frac
```

PCOD8047931

```
%BLOCK lattice_cart
3.066949  0.001744 -5.534685
0.004297  6.987767  0.007504
7.238800 -0.001000  5.255684
%ENDBLOCK lattice_cart
%BLOCK positions_frac
H   0.166971 -0.192714  0.482311
H  -0.142053  0.308850 -0.443914
H   0.010948  0.075867  0.461837
H   0.014652 -0.424265 -0.422768
H   0.021833  0.029773  0.248108
H   0.003539 -0.468975 -0.208830
H   0.258970  0.068585  0.409798
H  -0.233594 -0.430617 -0.370680
H  -0.409119 -0.232020  0.156789
H   0.434169  0.268840 -0.118818
H  -0.287830  0.022703  0.131319
H   0.313036 -0.476417 -0.092663
H   0.484409  0.100005  0.262280
H  -0.459750 -0.399510 -0.224211
H  -0.510393  0.037106  0.023437
H  -0.464010 -0.462833  0.014723
H  -0.318672  0.178108  0.447635
H   0.343802 -0.323859 -0.410171
H  -0.239384  0.066585 -0.352263
H   0.263472 -0.434532  0.389369
H  -0.138351 -0.022916 -0.192433
H   0.161962  0.476098  0.229637
H  -0.376595 -0.200311 -0.296900
H   0.400469  0.299142  0.334070
H  -0.470021  0.081183 -0.193188
H  -0.505780 -0.419864  0.230681
H   0.075513 -0.283990 -0.101524
H  -0.050399  0.214798  0.140547
H  -0.018146  0.486912  0.030767
H   0.042746 -0.012836  0.007500
H  -0.175785 -0.488728  0.161313
H   0.199949  0.012684 -0.123206
O   0.120267 -0.003589  0.413046
O  -0.094870  0.497214 -0.373100
O  -0.445567 -0.011575  0.125330
O   0.470605 -0.510982 -0.087173
O  -0.176505  0.173521 -0.484494
O   0.201653 -0.328522 -0.478157
O  -0.278708 -0.005907 -0.259514
O   0.302557  0.493308  0.296477
O   0.074373 -0.429759 -0.104851
O  -0.048737  0.069011  0.144133
O  -0.436445 -0.334207 -0.322604
O   0.461107  0.165695  0.360369
O  -0.051607  0.435024  0.131946
O   0.075423 -0.063589 -0.094076
O  -0.383080 -0.372899  0.176824
O   0.407938  0.127952 -0.138760
%ENDBLOCK positions_frac
```

207_1_4435

```
%BLOCK lattice_cart
7.195275 0.000016 0.000002
0.000016 7.192659 0.000004
0.000002 0.000004 7.189411
%ENDBLOCK lattice_cart
%BLOCK positions_frac
H  -0.386867  0.343619 -0.000001
H   0.387158 -0.350658  0.000001
H  -0.000000  0.386463 -0.347784
H   0.347137 -0.000427 -0.386978
H  -0.387158 -0.350661 -0.000000
H  -0.000001 -0.387429 -0.346527
H  -0.347136 -0.000425 -0.386978
H   0.347136 -0.000427  0.386977
H  -0.000002 -0.387428  0.346529
H   0.386867  0.343619 -0.000001
H  -0.347136 -0.000424  0.386975
H  -0.000002  0.386465  0.347784
H   0.500000  0.110170  0.152342
H   0.500001 -0.115947  0.153371
H   0.152930  0.496884 -0.113017
H   0.113160  0.152931  0.500002
H   0.500002 -0.115948 -0.153372
H   0.112833 -0.152619  0.500002
H   0.500001  0.110168 -0.152344
H  -0.152931  0.496881 -0.113018
H  -0.112831 -0.152616  0.500001
H  -0.113160  0.152931  0.500001
H   0.152930  0.496885  0.113016
H  -0.152932  0.496882  0.113016
O   0.500000  0.258453 -0.000001
O   0.500001 -0.265399  0.000001
O  -0.000001 -0.500842 -0.261929
O   0.261893  0.000779  0.500000
O   0.000000 -0.237926  0.500003
O   0.500002 -0.002644 -0.238099
O   0.238173  0.495581 -0.000000
O   0.500001 -0.002642  0.238098
O  -0.238174  0.495577 -0.000001
O  -0.000000  0.238002  0.500002
O  -0.000003 -0.500841  0.261930
O  -0.261892  0.000782  0.499998
%ENDBLOCK positions_frac
```

58_2_511

```
%BLOCK lattice_cart
12.596398 0.051273  0.037264
 -0.010725 4.049569 -0.015741
  0.020623 0.018489  6.329635
%ENDBLOCK lattice_cart
%BLOCK positions_frac
H   0.228320  0.446861  0.245418
H  -0.248222 -0.474824 -0.242374
H   0.253972 -0.051328  0.267934
H  -0.244321 -0.479584  0.234080
H   0.252290 -0.038441 -0.254926
H  -0.273709  0.025420 -0.271303
H   0.224831  0.463299 -0.231161
H  -0.272055  0.020996  0.251325
H   0.169672  0.258701 -0.036924
H  -0.191367 -0.276867 -0.046802
H   0.309672 -0.246617  0.463376
H  -0.328601  0.223838 -0.468107
H   0.122938  0.245230  0.314240
H  -0.115817 -0.260679 -0.372970
H   0.386932 -0.260753  0.135102
H  -0.140074 -0.270605  0.304778
H   0.356656 -0.247333 -0.185623
H  -0.406147  0.234770 -0.140545
H   0.093353  0.247282 -0.364733
H  -0.376415  0.228764  0.180179
H   0.479574 -0.465947 -0.000825
H  -0.020551 -0.052705  0.503287
H  -0.499229  0.034491 -0.005188
H   0.000091  0.446805  0.503921
O   0.183283  0.247762  0.205730
O  -0.203730 -0.273234 -0.205554
O   0.299427 -0.251977  0.304239
O  -0.199261 -0.279293  0.195541
O   0.296815 -0.239898 -0.293499
O  -0.318350  0.226834 -0.308766
O   0.179605  0.261641 -0.196397
O  -0.317091  0.222044  0.289279
O   0.047193  0.237493  0.504037
O  -0.067604 -0.261368  0.497300
O   0.432620 -0.256980  0.001970
O  -0.451901  0.242393 -0.007378
%ENDBLOCK positions_frac
```

169_2_7915

```
%BLOCK lattice_cart
 5.113806   8.999167 0.019509
-5.252810   9.003256 0.009643
-0.011739 -0.014666 4.538722
%ENDBLOCK lattice_cart
%BLOCK positions_frac
H   0.342132  0.292029  0.320432
H  -0.369819 -0.202325 -0.176967
H  -0.414701 -0.306572  0.155285
H   0.383164  0.398921 -0.345784
H   0.234660  0.439829 -0.012409
H  -0.264401 -0.350403  0.489444
H   0.478807 -0.088581  0.251131
H   0.495056  0.180348 -0.248678
H   0.341078 -0.447645  0.086471
H  -0.374116 -0.459368 -0.412138
H  -0.148589 -0.315882 -0.080128
H   0.122220  0.397284  0.420464
H   0.315474  0.215550  0.045601
H  -0.340007 -0.127771 -0.450878
H  -0.518184 -0.279495 -0.120179
H   0.486501  0.373167  0.379964
H   0.157156 -0.457980 -0.287584
H  -0.188982 -0.454337  0.214717
H  -0.485924  0.032616  0.158902
H   0.463361  0.056813 -0.341853
H   0.496863 -0.477384 -0.003753
H   0.469612 -0.428455 -0.503147
H  -0.026305 -0.472852 -0.171664
H  -0.002609 -0.447459  0.328000
O  -0.490890 -0.225094  0.025190
O   0.460436  0.317970 -0.474697
O   0.238915  0.516278 -0.141744
O  -0.271260 -0.425484  0.360210
O  -0.286816 -0.209004 -0.303936
O   0.260365  0.296560  0.191742
O   0.466271  0.110763 -0.161188
O   0.510635 -0.021299  0.338924
O  -0.472840 -0.430725 -0.323230
O   0.439607 -0.475458  0.175940
O   0.051609  0.495351 -0.491172
O  -0.080399 -0.414873  0.008093
%ENDBLOCK positions_frac
```

PCOD8036144

```
%BLOCK lattice_cart
3.919127 -4.880334  0.235701
1.875588  1.866014 -5.460710
5.536774  4.579005 -0.255672
%ENDBLOCK lattice_cart
%BLOCK positions_frac
H  0.135904  0.390126 -0.327021
H -0.153457 -0.375691  0.313380
H  0.007989 -0.265035 -0.494889
H -0.026080  0.279150  0.481522
H  0.073748 -0.293488 -0.261127
H -0.090746  0.307825  0.247718
H  0.365636 -0.263191 -0.439657
H -0.383940  0.275864  0.426817
H  0.398932  0.181451  0.256770
H -0.415112 -0.168881 -0.270198
H -0.469033  0.259568 -0.052963
H  0.453766 -0.246547  0.039275
H  0.216385  0.205711  0.087413
H -0.231769 -0.192744 -0.101227
H  0.350442 -0.141849  0.178333
H -0.365624  0.154897 -0.191898
H -0.439181 -0.440301 -0.342464
H  0.421490  0.453083  0.328774
H -0.299892 -0.218553  0.389168
H  0.282175  0.232665 -0.402898
H  0.068377 -0.037378 -0.167689
H -0.084354  0.051948  0.154372
H -0.139264  0.300055 -0.046178
H  0.124107 -0.285864  0.032599
O  0.117372 -0.325644 -0.381175
O -0.135348  0.339531  0.367686
O  0.363219  0.147368  0.133819
O -0.378639 -0.134514 -0.147531
O -0.473013 -0.260893 -0.437870
O  0.454761  0.273483  0.424684
O  0.142582  0.226795 -0.331427
O -0.160111 -0.212456  0.317927
O -0.361459  0.310419 -0.173669
O  0.346123 -0.297298  0.159929
O -0.034134  0.236434  0.070972
O  0.018759 -0.222126 -0.084345
%ENDBLOCK positions_frac
```

169_2_10608

```
%BLOCK lattice_cart
 4.393378  7.119216 -0.003994
-4.017702  7.052853 -0.005129
-0.001540  0.002506  5.117080
%ENDBLOCK lattice_cart
%BLOCK positions_frac
H -0.409014 -0.284717 -0.352267
H  0.400882  0.258688  0.147623
H  0.312054  0.409185  0.489269
H -0.320490 -0.434845 -0.010759
H -0.258784 -0.345563  0.317283
H  0.250680  0.320020 -0.182616
H  0.327834  0.047251  0.153493
H -0.335902 -0.073026 -0.346541
H  0.462715 -0.373657 -0.071858
H -0.471164  0.347929  0.427963
H  0.092996 -0.461195 -0.235040
H -0.101051  0.435844  0.264637
H  0.475759 -0.126636  0.428681
H  0.516241  0.100794 -0.071528
H  0.329194 -0.411207  0.207929
H -0.337356  0.385486 -0.292399
H -0.067822 -0.343738  0.057259
H  0.059626  0.318382 -0.442639
H  0.112270  0.137258  0.377394
H -0.120340 -0.162654 -0.122758
H  0.278706 -0.128180  0.199231
H -0.286854  0.102584 -0.301285
H  0.158973 -0.280188  0.023647
H -0.167101  0.254702 -0.476673
O  0.389453  0.174954  0.008327
O -0.397473 -0.200970 -0.491436
O -0.411158 -0.425076 -0.151435
O  0.402713  0.399227  0.348449
O  0.167865  0.411826 -0.321178
O -0.176019 -0.437183  0.178531
O  0.290858 -0.021084  0.287134
O -0.298955 -0.004480 -0.213188
O  0.282910 -0.298679  0.085352
O -0.291019  0.273074 -0.415163
O -0.011186 -0.283577 -0.057766
O  0.003082  0.258212  0.442187
%ENDBLOCK positions_frac
```

PCOD8047078

```
%BLOCK lattice_cart
 1.871528  0.031133 -3.760368
-0.103113  9.627472  0.029368
 7.102959 -0.011856  3.350493
%ENDBLOCK lattice_cart
%BLOCK positions_frac
H -0.470935 -0.481824  0.008758
H  0.462641  0.020798 -0.028432
H -0.460712 -0.336637  0.202536
H  0.454641  0.176787 -0.218585
H -0.151356  0.447036  0.199258
H  0.146574 -0.038438 -0.225922
H  0.351991  0.467741  0.184060
H -0.356454 -0.017576 -0.207616
H  0.517067 -0.096856  0.187660
H -0.527628  0.415686 -0.211569
H -0.299841  0.054345  0.200105
H  0.287598 -0.432594 -0.216173
H  0.199510  0.075304  0.226873
H -0.211165 -0.410769 -0.242273
H -0.177403 -0.231327  0.368767
H  0.169925  0.273580 -0.389870
H  0.329729 -0.238307  0.335100
H -0.336426  0.269024 -0.355282
H -0.000356 -0.336448 -0.395276
H -0.008580  0.155111  0.379228
H  0.001395 -0.148850 -0.476914
H -0.006445  0.347038  0.450636
H -0.042985 -0.184313 -0.239323
H  0.034824  0.299818  0.215192
O -0.447084 -0.483599  0.138541
O  0.442916  0.029262 -0.157831
O  0.498110  0.005808  0.159092
O -0.509321 -0.481791 -0.178883
O -0.467688 -0.240919  0.260863
O  0.461353  0.269874 -0.280700
O  0.024071 -0.228034  0.440580
O -0.031070  0.272968  0.538244
O -0.005907 -0.357623 -0.270610
O -0.005870  0.128645  0.255657
O -0.051355 -0.084965 -0.275396
O  0.046943  0.400465  0.247554
%ENDBLOCK positions_frac
```

PCOD8172143

```
%BLOCK lattice_cart
11.008881  0.009294 -0.003651
 -0.004573  3.991893  0.012244
  0.004025 -0.015182  5.062874
%ENDBLOCK lattice_cart
%BLOCK positions_frac
H -0.136685 -0.364083 -0.268365
H  0.128219 -0.391966 -0.262824
H -0.371916  0.125576  0.232533
H  0.363309  0.104834  0.237400
H -0.143289  0.135437 -0.337502
H  0.133801  0.108896 -0.334815
H -0.365921 -0.375000  0.161411
H  0.357360 -0.394340  0.165479
H -0.233415 -0.173184 -0.074363
H  0.225016  0.411930 -0.070507
H -0.275657  0.323610  0.425069
H  0.266408 -0.089288  0.429806
H -0.056790 -0.022816  0.058272
H  0.048059  0.261107  0.059836
H -0.452665  0.469365 -0.442638
H  0.443102 -0.244675 -0.440752
H  0.077419 -0.043172  0.392922
H -0.086871  0.283854  0.389580
H  0.413460  0.452506 -0.107060
H -0.422125 -0.224726 -0.111021
O -0.146857 -0.164981 -0.145346
O  0.138435  0.407606 -0.141405
O -0.362197  0.325872  0.354342
O  0.352971 -0.095426  0.359066
O -0.004425  0.118257  0.180275
O  0.495285 -0.388743 -0.320714
O -0.364231 -0.170917  0.039851
O  0.355472  0.401272  0.044282
O -0.145002  0.338687 -0.460097
O  0.135439 -0.095384 -0.456185
%ENDBLOCK positions_frac
```

152_2_118474

```
%BLOCK lattice_cart
 2.749962  5.015182 0.142688
-2.969020  4.885153 0.280579
 0.231039 -0.302749 5.926585
%ENDBLOCK lattice_cart
%BLOCK positions_frac
H -0.484807 -0.250952 -0.501763
H  0.413977  0.314724 -0.141141
H  0.224884  0.467067  0.195225
H -0.325010 -0.437165 -0.163253
H -0.286694 -0.284681  0.173617
H  0.249809  0.282748 -0.466758
H  0.432866 -0.031311  0.205428
H -0.492468  0.432345  0.159882
H -0.024620 -0.324925 -0.438153
H -0.364647  0.355335  0.472334
H -0.076783 -0.403088 -0.127506
H  0.385694  0.062090 -0.174291
H  0.197554 -0.153411  0.030358
H -0.275564  0.077711  0.333011
H  0.278254 -0.261242 -0.354198
H  0.002102  0.293608  0.386406
H -0.214080 -0.047787 -0.298740
H  0.028602  0.182678  0.000021
O -0.390410 -0.134460  0.286700
O  0.327705  0.513437  0.083761
O  0.112064  0.383286  0.413665
O -0.522271 -0.350907 -0.382067
O -0.175229 -0.484807 -0.051938
O  0.459917  0.165008 -0.254772
O  0.206790  0.014870  0.014993
O -0.248653  0.237619  0.345489
O -0.027272 -0.208320 -0.309770
%ENDBLOCK positions_frac
```

14_2_48453

```
%BLOCK lattice_cart
 4.274028 -0.495899 -0.007409
 0.693940  6.104765  0.009168
-0.074983  0.005107  6.682336
%ENDBLOCK lattice_cart
%BLOCK positions_frac
H  0.449924  0.236305  0.539071
H  0.423693 -0.185195  0.390492
H -0.049631  0.314337  0.038116
H -0.073919 -0.263766 -0.111119
H  0.444273  0.158535  0.308445
H  0.427854 -0.107452 -0.378686
H -0.058597  0.392138 -0.192714
H -0.063040 -0.341488  0.119543
H -0.357744  0.150084 -0.179172
H  0.242206 -0.100648  0.106709
H  0.140284  0.399484  0.320755
H -0.273931 -0.349699 -0.391646
H  0.258945  0.155070 -0.178647
H -0.374989 -0.103623  0.108616
H -0.242600  0.396945  0.320530
H  0.109429 -0.345007 -0.393645
O  0.447519  0.104885 -0.255550
O  0.434958 -0.054775  0.184694
O -0.053005  0.445498  0.243530
O -0.078810 -0.395455 -0.316209
O  0.449664  0.293200  0.396693
O  0.417874 -0.241958 -0.467209
O -0.048131  0.257569 -0.104188
O -0.069137 -0.206786  0.031111
%ENDBLOCK positions_frac
```

PCOD8045578

```
%BLOCK lattice_cart
 7.734411 -0.004397 -1.982279
-0.001970  4.250365  0.000625
 6.795719 -0.000229  3.376358
%ENDBLOCK lattice_cart
%BLOCK positions_frac
H  0.458991 -0.430581 -0.308536
H -0.489153 -0.429740  0.305209
H  0.458300  0.185532 -0.308084
H -0.488376  0.186366  0.305111
H  0.318358  0.378588  0.022135
H -0.350000  0.377628 -0.023765
H -0.289143  0.377840 -0.281309
H  0.258241  0.378458  0.279068
H -0.240760 -0.119546  0.277629
H  0.211191 -0.120922 -0.281583
H  0.010289 -0.311260  0.306334
H -0.040538 -0.312041 -0.309421
H  0.010349  0.072579  0.306712
H -0.040465  0.071776 -0.310024
H  0.149981 -0.120277 -0.023608
H -0.178890 -0.119390  0.019122
O  0.534822  0.377526 -0.311948
O -0.565464  0.377870  0.309548
O -0.065053 -0.119370  0.308921
O  0.036048 -0.119884 -0.313818
O -0.322001 -0.119833  0.221843
O  0.292833 -0.121186 -0.226351
O  0.175819  0.379229  0.224696
O -0.207129  0.378088 -0.226363
%ENDBLOCK positions_frac
```

11_2_15848

```
%BLOCK lattice_cart
-2.319854  0.939380 5.267056
 0.835730  5.812061 1.143502
-5.809818 -0.792117 0.710229
%ENDBLOCK lattice_cart
%BLOCK positions_frac
H  0.508875 -0.037294  0.046917
H -0.518974  0.672293  0.393696
H -0.359721  0.350162  0.210510
H  0.448464 -0.386182 -0.090943
H -0.091894 -0.020140 -0.088769
H  0.178250 -0.017680  0.197290
H -0.099774  0.475260 -0.066991
H  0.303655 -0.539899  0.071335
H  0.304968 -0.061978 -0.213405
H -0.244833  0.013412  0.235129
H  0.229340  0.437780 -0.230777
H -0.119863 -0.488931  0.355823
H -0.293483  0.176610  0.625710
H  0.038020 -0.504996 -0.471121
H  0.187971  0.104987 -0.344728
H -0.095733 -0.147925  0.460964
O -0.290188  0.044868  0.096462
O  0.374418 -0.089897  0.008098
O -0.327585  0.507982  0.244356
O  0.439326 -0.557115 -0.127630
O  0.229667 -0.067253 -0.337094
O -0.138403  0.016385  0.436419
O  0.084123  0.409276 -0.266745
O  0.021352 -0.447365  0.387203
%ENDBLOCK positions_frac
```

12_2_29187

```
%BLOCK lattice_cart
 6.151316 -0.027655 -0.035754
-2.042472  5.845221  0.026555
-1.978529 -2.930206  5.013556
%ENDBLOCK lattice_cart
%BLOCK positions_frac
H -0.223281  0.046076  0.453719
H  0.116269 -0.155813  0.437845
H  0.190796  0.128746 -0.230018
H -0.158971 -0.209775  0.225124
H  0.066811  0.421845 -0.189436
H -0.138581  0.430221  0.153632
H  0.376232 -0.199707  0.076134
H -0.412493  0.160907 -0.073837
H  0.095058 -0.252924  0.056225
H -0.092256  0.208899 -0.126989
H  0.226641 -0.100946 -0.186662
H -0.262349  0.062975  0.189124
O -0.011996  0.225767 -0.246008
O -0.023710 -0.272108  0.240340
O -0.322387 -0.068408  0.250959
O  0.286400  0.023605 -0.255622
O  0.182996 -0.274515 -0.058655
O -0.218790  0.229406  0.054395
%ENDBLOCK positions_frac
```

20_2_26425

```
%BLOCK lattice_cart
 7.356626 2.559948 0.030551
 4.253264 6.305892 0.011851
-0.016176 0.001573 3.176074
%ENDBLOCK lattice_cart
%BLOCK positions_frac
H  0.036928 -0.465693  0.119173
H -0.120067  0.446157 -0.383130
H -0.613872  0.208645 -0.393638
H  0.531080 -0.227547  0.103284
H  0.204627  0.091570  0.109395
H -0.287493 -0.110684 -0.387976
H  0.128580 -0.379214  0.427629
H -0.209342  0.358033 -0.073067
H  0.282987 -0.207769  0.464633
H -0.361522  0.184579 -0.033439
H  0.077616 -0.111200 -0.167669
H -0.153342  0.085178  0.332688
O -0.139504  0.427152 -0.089349
O  0.056636 -0.446229  0.412664
O -0.441797  0.066077 -0.211429
O  0.359048 -0.085215  0.285567
O  0.195383 -0.246391  0.592419
O -0.269726  0.219171  0.091624
%ENDBLOCK positions_frac
```

12_2_32449

```
%BLOCK lattice_cart
 4.906093 -2.166984 -1.768499
-3.225085  3.887306 -2.634777
 3.832468  1.696101  2.815830
%ENDBLOCK lattice_cart
%BLOCK positions_frac
H -0.577331 -0.194618 -0.311939
H  0.570481  0.168518  0.197454
H -0.067852  0.264434 -0.348492
H  0.043682 -0.311409  0.227428
H -0.215193 -0.383900  0.491248
H -0.493891 -0.008725  0.481110
H  0.231404  0.334822 -0.126568
H -0.256366 -0.380865 -0.005929
H -0.458266 -0.159511  0.056927
H  0.369843  0.172317 -0.104713
H -0.050212 -0.066160 -0.078309
H  0.197935  0.342681 -0.605366
O  0.156218  0.395290 -0.383508
O -0.178719 -0.439869  0.267773
O -0.445277  0.020649 -0.324495
O  0.431107 -0.051465  0.202261
O  0.226932  0.248658  0.010101
O -0.242547 -0.282667 -0.134704
%ENDBLOCK positions_frac
```

15_2_201714

```
%BLOCK lattice_cart
 1.192347 -1.336676  3.388231
 0.882875  6.031658 -0.189029
-6.038371 -0.064357  0.034045
%ENDBLOCK lattice_cart
%BLOCK positions_frac
H  0.161906 -0.138648 -0.250602
H -0.280846  0.027596  0.295186
H -0.111129  0.371262  0.283682
H  0.331498  0.204932 -0.262007
H  0.181252  0.406708  0.007755
H  0.570791 -0.108305 -0.277763
H -0.130411 -0.174085  0.025563
H -0.520018  0.340922  0.310938
H  0.408630 -0.358122  0.228753
H  0.019258 -0.098549  0.411450
H -0.357684 -0.409182 -0.195513
H  0.031414  0.331021 -0.378333
O  0.202234  0.470861  0.178149
O  0.344579 -0.054575 -0.330526
O -0.151382 -0.238174 -0.144889
O -0.293975  0.287031  0.363431
O  0.267916  0.352267 -0.232633
O -0.217342 -0.119770  0.265810
%ENDBLOCK positions_frac
```

84_2_1419

```
%BLOCK lattice_cart
4.212594 -0.029181  0.000138
0.008311  4.213037 -0.000045
0.000235 -0.000071  7.499247
%ENDBLOCK lattice_cart
%BLOCK positions_frac
H  0.103352 -0.029565 -0.000001
H -0.008223 -0.059066 -0.500001
H  0.489890 -0.028822 -0.000001
H -0.003580 -0.445216 -0.500002
H  0.331110  0.296249  0.392275
H  0.405262 -0.376787  0.164890
H  0.405259 -0.376785 -0.164886
H -0.346283 -0.370906 -0.333777
H  0.331102  0.296248 -0.392256
H -0.254517  0.306818 -0.107520
H -0.346267 -0.370903  0.333769
H -0.254515  0.306814  0.107515
O  0.199671  0.233915 -0.499994
O  0.297268 -0.172515  0.000001
O -0.149621 -0.255634 -0.500009
O -0.188200  0.177552 -0.000001
O -0.467380  0.485190  0.249100
O -0.467389  0.485201 -0.249100
%ENDBLOCK positions_frac
```

20_2_28176

```
%BLOCK lattice_cart
 3.418243 5.235012 -0.004297
-3.428522 5.250172 -0.022436
 0.000972 0.362817  3.962550
%ENDBLOCK lattice_cart
%BLOCK positions_frac
H -0.017492  0.460717 -0.085618
H  0.012203 -0.478739  0.404517
H -0.473021  0.480449 -0.459456
H -0.506536  0.448042  0.151839
H -0.159101  0.358999  0.207342
H  0.214077 -0.374809 -0.244645
H  0.206951  0.125334  0.107978
H -0.149874 -0.232287 -0.417511
H -0.383610  0.133515  0.483439
H  0.349328 -0.239156 -0.062217
H  0.453572 -0.036704  0.285802
H  0.514293 -0.007736 -0.224080
O -0.000072  0.358929  0.130279
O -0.001880 -0.381120 -0.396162
O -0.399506  0.373820  0.345636
O  0.368018 -0.393184 -0.153058
O -0.383528 -0.024985  0.559425
O  0.355719 -0.022976  0.087050
%ENDBLOCK positions_frac
```

2_2_342692

```
%BLOCK lattice_cart
 0.368881 -2.911415  0.057559
 1.833515  0.322898  4.525229
-4.548334 -0.558160  1.889219
%ENDBLOCK lattice_cart
%BLOCK positions_frac
H  0.044896  0.098569  0.054520
H  0.059712  0.025398 -0.445547
H -0.290820 -0.168255  0.253127
H  0.393304  0.291847 -0.246016
H -0.147691  0.329119  0.252035
H  0.250152 -0.205664 -0.247834
H -0.444610 -0.474679 -0.448771
H -0.451796 -0.401408  0.051096
O -0.022226  0.141384  0.247480
O  0.125021 -0.017838 -0.252682
O  0.529194 -0.522451 -0.253412
O -0.426467 -0.354032  0.246384
%ENDBLOCK positions_frac
```

SAMPLE CASTEP INPUT FILES

207_1_4435.param

```
task              : geometryoptimization
xc_functional     : PBE
basis_precision   : fine
opt_strategy      : speed
geom_method       : BFGS
finite_basis_corr : 0
```
