# Supplementary material for: Mapping uncharted territory in ice from zeolite networks to ice structures
Source: Nat Commun. 2018 Jun 5;9:2173. doi: 10.1038/s41467-018-04618-6 (PMC5988809; doi:10.1038/s41467-018-04618-6)
Supplement: Supplementary file 3 — Description of Additional Supplementary Files [file 41467_2018_4618_MOESM3_ESM.pdf]

Description of Additional Supplementary Files

File Name: Supplementary Data 1
Description: Structure data for the most promising proposed novel ice candidate structures, and sample Castep input file to facilitate reproduction of the density-functional-theory geometry optimisations.
